# Constrained instruments and their application to Mendelian randomization with pleiotropy

**DOI:** 10.1002/gepi.22184

**Published:** 2019-01-12

**Authors:** Lai Jiang, Karim Oualkacha, Vanessa Didelez, Antonio Ciampi, Pedro Rosa‐Neto, Andrea L. Benedet, Sulantha Mathotaarachchi, John Brent Richards, Celia M. T. Greenwood, and for the Alzheimer’s Disease Neuroimaging Initiative

**Affiliations:** ^1^ Lady Davis Institute for Medical Research, Jewish General Hospital Montreal Quebec Canada; ^2^ Department of Epidemiology, Biostatistics and Occupational Health and Gerald Bronfman Department of Oncology McGill University Montreal Quebec Canada; ^3^ Department of Mathematics, Université du Québec à Montréal Montreal Quebec Canada; ^4^ BIPS & Department of Mathematics Leibinz Institute for Prevention Research and Epidemiology, University of Bremen Bremen Germany; ^5^ Department of Neurology & Neurosurgery McGill University Montreal Quebec Canada; ^6^ Translational Neuroimaging Laboratory, McGill University Research Centre for Studies in Aging Douglas Hospital, McGill University Montreal Quebec Canada; ^7^ Department of Medicine McGill University Montreal Quebec Canada

**Keywords:** instrumental variables, Mendelian randomization, pleiotropy, smoothed algorithm

## Abstract

In Mendelian randomization (MR), inference about causal relationship between a phenotype of interest and a response or disease outcome can be obtained by constructing instrumental variables from genetic variants. However, MR inference requires three assumptions, one of which is that the genetic variants only influence the outcome through phenotype of interest. Pleiotropy, that is, the situation in which some genetic variants affect more than one phenotype, can invalidate these genetic variants for use as instrumental variables; thus a naive analysis will give biased estimates of the causal relation. Here, we present new methods (constrained instrumental variable [CIV] methods) to construct valid instrumental variables and perform adjusted causal effect estimation when pleiotropy exists and when the pleiotropic phenotypes are available. We demonstrate that a smoothed version of CIV performs approximate selection of genetic variants that are valid instruments, and provides unbiased estimates of the causal effects. We provide details on a number of existing methods, together with a comparison of their performance in a large series of simulations. CIV performs robustly across different pleiotropic violations of the MR assumptions. We also analyzed the data from the Alzheimer’s disease (AD) neuroimaging initiative (ADNI; Mueller et al., 2005. Alzheimer's Dementia, 11(1), 55–66) to disentangle causal relationships of several biomarkers with AD progression.

## 1 INTRODUCTION

**Figure 1:**
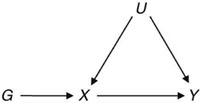
A directed acyclic graph representing a situation where Mendelian randomization using genetic variants **G** as instruments can be useful for inferring whether a phenotype **X** is causally related to an outcome **Y**. **U** represents unmeasured confounding factors

**Figure 2:**
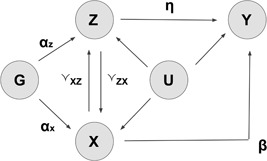
A general diagram representing potential pleiotropic influences in Mendelian randomization studies. α_x_, α_z_: genetic association parameters between **X**~**G** and **Z**~**G**; *β*: causal effect of interest (X on Y); γ_*xz*_ and γ_*zx*_: possible direct causal effects of **X** on **Z** and **Z** on **X**, respectively; *η*: the pleiotropic pathways of **Z** on **Y**; **G**: genotypes; **X**: phenotype of exposure; **Y**: response of interest; **Z**: potential pleiotropic phenotypes

Mendelian randomization (MR) is a popular epidemiological study design that incorporates genetic information (**G**) as an instrument to estimate the causal effect of a modifiable exposure (**X**) on a disease (**Y**; Figure 1). From a statistical perspective, MR is an application of instrumental variable methods (Didelez & Sheehan, 2007; Lawlor, Harbord, Sterne, Timpson, & Davey Smith, 2008; Smith & Ebrahim, 2003; Wehby, Ohsfeldt, & Murray, 2008) to eliminate bias from unmeasured confounding factors (**U**). Assuming a structural model set‐up, the following conditions are necessary for **G** to be a valid instrument: (A1) **G** and **X** are not independent; (A2) **G** and **Y** are conditionally independent given exposure **X** and unmeasured confounding factors **U**; (A3) **G** and confounders **U** are independent. MR is complicated by the possible violation of these assumptions; perhaps one of the most important cases is the possible presence of pleiotropy. Pleiotropy occurs when more than one phenotype is influenced by the same group of genotypes. If these phenotypes are found on the causal pathway for the response **Y**, then pleiotropy is a violation of assumption (A2). However, it is possible to accommodate pleiotropy within an extension of the original causal framework that includes one or more additional phenotypes (**Z**; Figure 2).

In econometric research, a framework like Figure 2 has been discussed to account for multiple risk factors simultaneously (Angrist, 2006; Ludwig & Kling, 2007; Wooldridge, 2015). However, in genetic studies the extraction of causal effects for individual risk factors with pleiotropic phenotypes is rarely discussed (Burgess & Thompson, 2015; Kang, Zhang, Cai, & Small, 2016). Instead, most of the recent MR studies are conducted using the two‐stage least squares (2SLS) estimator approach (Baum, Schaffer, & Stillman, 2003). Specifically, a prediction of **X** is constructed from the ordinary least square (OLS) regression **X**∼**G** and called $\hat{X}$. Then the OLS regression $Y\sim \hat{X}$ is fit and the slope $\hat{\beta }$ is proposed as the causal effect estimator.

When there are exogenous variables, they can be included as covariates. Hence a potential extension of *2SLS* to accommodate pleiotropy is to control for **Z** as covariates. This, however, is unsatisfactory. It would be ideal to identify instruments, **G**, for **X** that are unrelated to **Z** to estimate the causal effect of **X** on **Y** in the absence of pleiotropic effects. However, adjusting for covariates, **Z**, will enable construction of an instrument for **X**|**Z**, which is not answering the same question. Furthermore, controlling for **Z** can induce collider bias (Cole et al., 2009; Greenland, 2003). It is worth noting that adjusting for **Z** as covariates in *2SLS* is equivalent to estimating the causal effect on measures that have been residualized for **Z**. That is, to work with (**G** ^∗^, **X** ^∗^, **Y** ^∗^), where **G** ^∗^, **X** ^∗^, and **Y** ^∗^ are defined as **G** ^∗^ = (I −* P_z_*)**G**, **X** ^∗^ = (I − *P_z_*)**X**, and **Y** ^∗^ = (I − *P_z_*)*Y*, where *P_z_* = **Z^T^**(**Z^T^Z**)**^−1^Z** (Lovell, 2008; Wang & Zivot, 1998). More details can be found in Appendix A.

A second approach to coping with pleiotropy is based on the multiple linear regression of **Y** on **X** and **Z** jointly. However, if **X** and **Z** are highly correlated, the resulting estimator of *β* could be unstable, that is, the standard errors could be large (Farrar & Glauber, 1967; Grapentine, 2000; Grewal, Cote, & Baumgartner, 2004). Collider bias is also a concern here.

In a third approach, Some Invalid Some Valid Instrument Variable Estimator (*sisVIVE*; Kang et al., 2016), pleiotropy is treated as unobservable and **G** is modeled as a mixture of “valid” and “invalid” instruments, with an *L*_1_ penalized regression to infer the causal effect of **X** on **Y**. This approach is not guaranteed to eliminate pleiotropy: indeed, the *sisVIVE* estimator $\hat{\beta }$ would be biased when more than 50% of genotypes are pleiotropic. Moreover, if the *α*_*x*_ are much stronger than *α*_*z*_ (Figure 2) then *sisVIVE* may have difficulty identifying the pleiotropic genotypes, which would give biased causal effect estimates.

A variety of additional approaches to coping with pleiotropy can be found in the literature, for example, direct genotype selection, generalized methods of moments (GMM), Egger regression, and so forth. However, there has been limited recent work on solutions for inference when potential pleiotropic phenotypes are observed.

In this paper we present a novel approach to dealing with pleiotropy, based on the general framework of Figure 2: The idea is to construct a new instrumental variable by maximizing the association with **X** (i.e., instrumental strength) and minimizing possible correlation with potential pleiotropic phenotypes **Z**. There are three innovative aspects in this method: (a) the pleiotropic effect is eliminated by shrinking the correlation with potential pleiotropic phenotypes toward zero; (b) the instrumental strength is retained coherently in the model; (c) our penalization algorithm forces approximately sparse and valid genotype selection, which reduces the overfitting problem resulting from the use of multiple genotypes, especially when the number of genotypes is larger than the number of samples where most existing IV methods fail.

After introducing notation and outlining a formal framework for pleiotropy that also accommodates existing research (Section 2), we devote Section 3 to the presentation of our novel idea: constrained instrumental variable (CIV). A computationally feasible method, *CIV_smooth*, is then introduced to implement instrument construction and causal effect estimation. In Section 4 we compare by simulation the performance of our methods with the closest popular approaches, including variants of *2SLS* approach, *sisVIVE* and *Allele* scores. In Section 5 we conduct an MR study estimating the effects of four biomarkers (amyloid β [Aβ] 1–42, total tau protein [Ttau], phosphorylated tau protein [Ptau], and fluoro‐D‐glucose uptake [FDG_SUVR]) on Alzheimer’s disease (AD) risk using our methods.

## 2 NOTATION AND BACKGROUND

### 2.1 Notation

For each individual, *i* = 1, *…n*, let *Y*_*i*_ denote the response of interest, and **Y** = (*Y*_1_, *…*,*Y*_*n*_)^T^ ∈ *R* ^*n* × 1^ the vector of observations. Let **G** *_**i**_* ∈ *R* ^*p*^ represent the set of genotypes, where *p* is the number of single nucleotide polymorphisms (SNPs) being analyzed, and **G** = (**G**_**1**_, *…*, **G** *_**n**_*)^T^ ∈ *R* ^*n* × *p*^ the matrix of observations. Also, we denote by **Z** *_**i**_* ∈ *R* ^*k*^ the vector of additional phenotypes that may be affected by some elements of **G**_*i*_, and by **Z** = (**Z**_**1**_ *, …,* **Z** *_**n**_*)^T^ ∈ *R* ^*n* × *k*^ the matrix of these observations. Finally, let **X** = (*X*_1_ *, …, X*_*n*_)^T^ ∈ *R* ^*n* × 1^ denote the vector of the phenotype of interest.

Figure 2 lays out a general structure for our explorations. We assume that genotype **G**, phenotype of interest **X**, the response **Y**, and potential pleiotropic phenotypes **Z** have all been measured for each individual. The *total causal effect* of **X** on **Y** is the sum of the *direct causal effect* represented by the scalar parameter *β*, and any *indirect causal effects*. The latter is a product of the causal effect of **X** on **Z**, represented by *γ*_*xz*_, and the direct causal effect of **Z** on **Y**, represented by *η*. Pleiotropy is present when the association between **G** and **Z**, represented by the parameter *α*_*z*_, is nonzero. In this case, as previously mentioned, conditioning on **Z** may induce collider bias. The methods discussed below attempt to address this issue.

The genetic variants in **G** are strong instruments for **X** if the association between **G** and **X**, represented by *α*_*x*_, is strong. Note that **G** may contain many genetic variants and only some of them may influence **Z**. The relationships between phenotype of interest **X**, potential pleiotropic phenotypes **Z**, unmeasured confounders **U**, and outcomes **Y** may vary from one situation to another, that is, not all of the edges or arrows in Figure 2 need to be present in every particular study or scenario.

The relationships in Figure 2 can be formally expressed in the following linear structural equations:

(1a) $$Z=G\alpha_{z}+\zeta_{z}U+\varepsilon_{z},$$

(1b) $$X=G\alpha_{x}+Z\gamma_{zx}+\zeta_{x}U+\varepsilon_{x},$$

(1c) $$Y=X\beta +Z\eta +\zeta_{y}U+\varepsilon_{y},$$

Or

(2a) $$X=G\alpha_{x}+\zeta_{x}U+\varepsilon_{x},$$

(2b) $$Z=G\alpha_{z}+X\gamma_{xz}+\zeta_{z}U+\varepsilon_{z},$$

(2c) $$Y=X\beta +Z\eta +\zeta_{y}U+\varepsilon_{y}.$$

The parameters *ζ*_**x**_, *ζ*_**z**_, and *ζ*_**y**_ represent the impact of unmeasured confounding factors **U** on **X**, **Z**, and **Y**, respectively. The errors *ε*_*x*_, *ε*_*z*_, and *ε*_*y*_ for **X**, **Z** and **Y**, respectively, are assumed to be independent and identically distributed.

Each of the following assumptions for the relationship between **X** and **Z** represents an interesting scenario.

(i) **X** and **Z** are conditionally independent given **G** and **U** (*γ*_*zx*_ = *γ*_*x*_ *_z_* = 0), that is the simplest case of pleiotropy.
(ii) There is a direct causal impact of **Z** on **X** (*γ*_*zx*_ ***≠*** 0 and *γ*_*xz*_ = 0).
(iii) There is a direct causal impact of **X** on **Z** (*γ*_*xz*_ ≠ 0 and *γ*_*zx*_ = 0). In this case, the total causal effect of **X** on **Y** is *β* + *γ*_*xz*_ *η*. Although this is an important scenario, we do not address it in this paper, since we are focusing on the estimation of *β*.

Assuming that the set **G** may be quite large, containing many genetic variants in association with **X**, using all elements of **G** in the analysis may introduce bias in the estimation of *β*: Indeed some components of **G** may affect **Z**, so that the total causal effect from **G** to **Y** is mediated by both **X** and **Z**. In MR applications, those components of **G** which do have an impact on **Z** are usually eliminated, possibly causing other types of bias (e.g., weak instrument bias, Burgess, Thompson, & Collaboration, 2011; selection bias, Smith & Ebrahim, 2003; etc.).

Our goal is to determine the best MR estimator of *β* when possibly pleiotropic variables **Z** are measured and available and when the set **G** contains several variants. We are searching for the best approach to remove bias in the estimation of *β*.

### 2.2 Two‐stage least squares

The simplest MR method is *2SLS* regression. Given valid instruments **G**, the following two stages define a *2SLS* model:

1. In the first stage, a new variable $\hat{X}$ is obtained from the fitted values from OLS regression **X**∼**G**.
2. In the second stage, the OLS estimates of *β* from the regression **Y**∼ $\hat{X}$ are obtained.

*2SLS* works well if **G** is a set of valid instruments with *α*_*z*_ = 0. This rarely occurs naturally, but can sometimes be achieved by carefully selecting a subset of variants **G** that are approximately valid. Most researchers using MR make intensive efforts to select instruments **G** that are most likely to satisfy the three key assumptions (A1–A3) of MR. Variants known to be in pleiotropic pathways, or variants showing associations with possibly pleiotropic phenotypes, are removed from the set of variants to be considered. However, this selection process is necessarily ad hoc. In this paper, we refer to the original *2SLS* as “*2SLS_naive*.”

A variation of the *2SLS* approach—the *Allele* score method Burgess and Thompson (2013) constructs summarized genetic scores **G** ^∗^ = **Gw**. The weights **w** correspond to estimated genetic effect sizes for each genotype, and can be derived internally from data under analysis or externally from prior knowledge. Protection against winner’s curse can be incorporated into the estimation of **w** through internal cross‐validation or external sources for the estimates of genetic associations.

### 2.3 Statistical methods for selection of valid instruments

Several methods have been proposed for improving causal estimation in the presence of pleiotropy, for example, Egger regression (Bowden, Smith, & Burgess, 2015), *CUE* (Davies et al., 2015), *LIML* (Hansen, Heaton, & Yaron, 1996), *Allele* score (Burgess & Thompson, 2013); these methods generally assume that the pleiotropic phenotypes are unknown, and use all the components of **G**.

In the same vein, Kang et al. (2016) proposed to select components of **G**, again without explicitly using the phenotypes **Z**. The proposed approach, named by the authors as *“some invalid some valid IV estimator* *(sisVIVE)*,” incorporates all causal effects from **G** to **Y** using the following model:

(3a) $$Y_{i}=G_{i}\delta +X_{i}\beta +\varepsilon_{y}i,$$

(3b) $$E(\varepsilon_{y}i|G_{i})=0,i=1\ldots n,$$

(3c) $$X_{i}=G_{i}\alpha +\varepsilon_{x}i,$$

where ***δ*** represents the direct effects of the instruments **G** on outcome **Y**. Indirect effects of **G** on **Y** are captured through **X**, and ***β*** represents the causal effect parameter of interest. *α* is the association parameter between **G** and **X**. The central idea of Kang et al. (2016) is to operate a sparse selection of genetic variants (components of **G**) by a LASSO type penalization, which leads to the constrained optimization problem:

$$(\beta ,\delta )\in argmin1/2||P_{G}(Y-G\delta -X\beta )|{|}_{2}^{2}+\lambda ||\delta |{|}_{1},$$

where **P**_**G**_ **= G**(**G** ^T^ **G**)^−**1**^ **G** ^T^. In other words, the projected error of predicting **Y** from **G** and **X** is minimized, while controlling the impact of invalid instruments in **G** on **Y** (through the penalty term). It has been shown that, under certain conditions, *sisVIVE* is robust to certain types of invalid instruments, for example, pleiotropic genotypes and their direct causal effect on **Y** (without going through **X**).

### 2.4 Adjustment for exogenous or endogenous variables

In MR terminology, the term “endogenous variable” describes factors that are explained by the genotype–phenotype relationships and impact response **Y**. For example, common endogenous variables include health‐related behaviors and risk‐related phenotypes. Both **X** and **Z** in Figure 2 are endogenous as they are determined by genotypes and have impact on the response, albeit with different functions. Endogenous variables are the variables of primary concern for MR studies.

In contrast, covariates such as age and sex that are not associated with the genotype–phenotype causal pathways of interest are termed “exogenous”; normally it is possible to adjust for these variables in a straightforward way. One solution is to replace **G**, **X**, **Y** by **G** ^∗^ = (**I** − **P**_**z**_)**G** and **X** ^∗^, **Y** ^∗^, respectively, where **P**_**z**_ **= Z** ^T^(**Z** ^T^ **Z**)^−**1**^ **Z**. Then straightforward *2SLS* can be applied to the new (**G** ^∗^, **X** ^∗^, **Y** ^∗^); we refer to this method as “*2SLS_exo*.” It is worth noting that this method is equivalent to controlling for **Z** as covariates in both first stage and second stage regressions. Details are given in Appendix A.

Although this has sometimes been implemented with pleiotropic phenotypes, this is not an appropriate approach: the estimate of *β* remains biased, as pleiotropic variables, **Z**, are not exogenous (Engle, Hendry, & Richard 1983). Note, if *α*_*z*_ ≠ 0, treatment of **Z** as an exogenous variable may introduce collider bias due to dependence between **G** and **u** after conditioning on **Z**.

Another multiple regression‐type approach, this time to account for endogenous variables, can be derived from the underlying linear structural model (Figure 2) by building a multiple linear regression of **Y** on $\hat{X}$ and $\hat{Z}$ jointly in a *2SLS* model, where $\hat{X}$ and $\hat{Z}$ are the predicted phenotypes using **G** as the instruments. We refer to this method as “*2SLS_mul*.” The *2SLS_mul* method uses **G** to account for endogeneity of **Z**, without controlling for it explicitly. However, using this approach, the resulting estimator of *β* will be unstable if **X** and **Z** are highly correlated (Farrar & Glauber, 1967; Grapentine, 2000; Graham, 2003; Grewal et al., 2004).

Both of these two multiple regression solutions for secondary phenotype variables can be embedded within the *Allele* score method to adjust for **Z** variables, and we refer to these methods as “*Allele_mul*” and “*Allele_exo*.” The original *sisVIVE* approach assumes all pleiotropic phenotypes are unmeasured and treats them as sources of the indirect causal effect of **G** on **Y**, and thus does not use **Z** variables directly. If measures of secondary phenotypes are available, Kang et al. (2016) suggested adjusting (**G**, **X**, **Y**) a priori on **Z** (as in *2SLS_exo*), thus treating **Z** measures as exogenous variables. We refer to this method as “*sisVIVE_exo*.”

### 2.5 Design choices: One sample, a first sample with and without an external validation sample, or two sample

In the causal inference literature, the term “*one‐sample analysis*” refers to the situation in which a single data set (a sample) is used to perform a data analysis task, typically the estimation of the parameters in a model. Unless the sample size is enormous, a one‐sample analysis is often considered flawed due to the problem of overfitting; see Thaler’s work on “winner’s curse” (Thaler, 1988). Using sample splitting techniques—that is cross‐validation or splitting the sample into a “learning sample” and a “testing or validation” sample (James, Witten, Hastie, & Tibshirani, 2013) will alleviate the problem, though not remove it. Obtaining an external validation sample, that is, a second sample from an external source with exactly the same variables as the original one, has been argued to be a better approach to reduce overfitting bias and increase generalizability (Friedman, Hastie, & Tibshirani, 2001).

In the framework of this paper, the model presented in Figure 2 has the parameters of interest *α*_*x*_ and *β*. In Table 1 we distinguish different study designs that could be used with pleiotropic phenotypes **Z**. In the one sample setup (first row in Table 1) the data includes variables **G**, **X**, **Z**, and **Y**. An external validation sample, if available, must also contain **G**, **X**, **Z**, and **Y** (second row of Table 1). We will refer to this situation as “one sample analysis with external validation,” to distinguish it from both the “one sample” and the “two‐sample” setups. Note that we consider one‐sample designs with internal splitting as a one‐sample situation.

**Table 1:** A comparison of study designs considered here

Study design	Number of samples	Variables required
One sample analysis	One	(X, Z, Y, G) on 1 data set.
One sample analysis with external validation sample	Two: (1) learning and (2) validation	(X, Z, Y, G) on both data set 1 and data set 2.
Two‐sample analysis in Mendelian randomization	Two: (1) Learning weights and (2) learning causal effects	(X, Z, G) on data set 1. (G, Y) on data set 2.

Indeed in the causal inference literature, the “two‐sample” setup is a design in which two studies are performed for two distinct analytic tasks. Specifically, in one study given **G** and **X**, *α*_*x*_ is estimated; and in the other study given **G** and **Y** (or **G**, **X**, and **Y**), *β* is estimated. One advantage to this approach is that large datasets (Study 1) may be available to estimate the instrument strengths even though **Y** was not measured. Another advantage is that such separation of the data protects against overfitting. If valid instruments are constructed using the data set with **G** and **X**, the corresponding causal effect estimates from the second sample should be less subject to the overfitting bias.

Although all methods discussed in this paper work for the one‐sample set‐up, not all of these methods can be adapted to the one sample analysis with external validation or the two‐sample set‐up. The ordinary *2SLS* method adapts easily to a two‐sample set‐up (Angrist & Krueger, 1992; Dee & Evans, 2003). However, this adaptation does not improve asymptotic efficiency (Inoue & Solon, 2010). When there are additional phenotypes (**Z**) to be considered, neither *2SLS_exo* nor *2SLS_mul* can be adapted to one sample with external validation set‐up or the two‐sample set‐up because they both require one‐sample individual level data to calculate the appropriate residuals. In contrast, methods that propose valid instrument construction using **Y** include *sisVIVE*, and its variants (*sisVIVE_exo* and *sisVIVE_mul*) can be extended to one sample analysis with external validation. Specifically, the valid instrument selection is obtained from the original sample, and is used to infer the causal effect *β* on the external validation sample. Furthermore, the *Allele* method and its variants adjusting for exogenous variables (*Allele_exo* and *Allele_mul*) extend to the two‐sample situation as the *Allele* weights only depend on **G**, **X** (and **Z**).

## 3 CONSTRAINED INSTRUMENTAL VARIABLE (CIV) METHODS

Let us consider the situation where potentially pleiotropic phenotypes (**Z**) are measured and available. We propose here a novel approach that we call “CIV”. The central idea is to maximize instrument strength, whereas attempting to control the impact of pleiotropic effects. In what follows we will consider two cases separately. In Section 3.1, we show that in the particular case *p* < *n*, a new instrumental variable can be obtained as a solution of an unpenalized maximization problem. In Section 3.2, we show that the addition of an appropriate penalty term to the aforementioned maximization problem leads to workable solutions with no restriction on *p*.

### 3.1 *CIV_naive*:* CIV* when *p* < *n*

We are looking for a linear combination of the genotype data such that the resulting instrument strength is maximized, and the association between new instruments and pleiotropic phenotypes **Z** is zero. In mathematical terms we aim to find a vector **c** ∈ *R* ^*p*^ which solves the following optimization problem:

(4) $$\begin{array}{l} \max\;c^{T}G^{T}X\;\\ c\;\in \;R^{p} \end{array}$$

subject to conditions:

(5a) $$c^{T}G^{T}\text{Gc}=1,$$

(5b) $$c^{T}G^{T}Z=0.$$

Note that Equation (5a) is a normalizing condition which ensures the unicity of the solution (the norm of the projection **c** on **G** is constrained to be 1).

This maximization problem is well‐defined when *p* < *n* and *p* ≥ *k* (where *k* is the number of possibly pleiotropic phenotypes), and can be solved using simple linear algebra (see Appendix B). Let $\hat{c}$ be the solution to the constrained optimization problem above. We will refer to $G\hat{c}$ as the *CIV_naive* instruments.

The strength of the *CIV_naive* instruments can be measured by the *F*‐statistic of linear model **X**∼**G** against the null hypothesis that the excluded instruments are irrelevant. As a rule of thumb, instrumental variables with *F*‐statistics < 10 are usually considered weak instruments. *CIV_naive* is designed to retain instrument strength, however, it may not always yield the strongest possible global *F*‐statistic due to constraint (5b; Boyd & Vandenberghe, 2004; Tofallis, 1999).

In a one‐sample analysis, the new instrumental variable **Gc** is used to infer the causal effect of **X** on **Y** using methods from linear structural equation modeling such as *2SLS*. Furthermore, the *CIV_naive* approach translates naturally to two‐sample analyses. The linear vector **c** is estimated in the first‐stage data set, and the estimate $\hat{c}$ is used in the second‐stage data set to create the new instrumental variable $G\hat{c}$ and to estimate the causal effect *β*.

### 3.2 A penalized maximization: *CIV_smooth*

The existence of a unique solution for the optimization problem when *p* < *n* (see Section 3.1 and Appendix B) is a definite asset of *CIV_naive*. In contrast, an important concern is that when *p* > *n*, solutions may not exist. Another concern is that, regardless of whether or not *p* < *n*, a reduction in the number of components may be desirable to avoid overfitting and to provide insight into the causal impact of SNPs. To address these two concerns, we propose an improvement of *CIV_naive* which guarantees existence (though not uniqueness) of solutions and allows for variable selection. This is achieved by imposing a penalty on the optimization problem (4). We will call the proposed method *CIV_smooth*.

**Figure 3:**
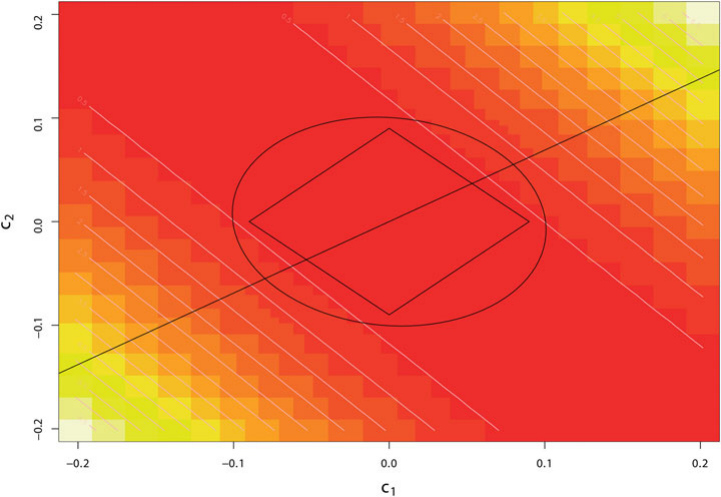
Graph demonstrating the maximization problem with LASSO (L_1_) penalty and L_2_ penalty. Rectangle: LASSO penalty contour with the same level of penalization. Circle: L_2_ penalty contour with the same level of penalization. Straight line: the solution space required by condition (5b); it has zero probability of intersecting a sparse solution here. Pixels with color from yellow to red: co‐ordinates of C = (c1, c2) with absolute correlation values from high to low levels

Different choices of penalty functions lead to different solutions. However, in this context, neither *L*_1_ nor *L*_2_ penalties will result in a sparse solution under any level of regularization, because of the linear constraint (3c). Figure 3 illustrates this for two instruments **G** = (**g**_**1**_, **g**_**2**_); the *L*_1_ and *L*_2_ penalty contours intersect the linear constraint at nonsparse solutions for **c**.

Therefore, we propose instead to use a *L*_0_ penalty, and to maximize the constrained function

(6) $$\begin{array}{l} \max c^{T}G^{T}{X‐\lambda |c|}_{0}\\ c\;\in \;R^{p} \end{array}$$

subject to conditions:

(7a) $$c^{T}G^{T}\text{Gc}\leq 1,$$

(7b) $$\begin{array}{l} c^{T}G^{T}Z=0, \end{array}$$

where |**c**|_0_ is the *L*_0_ norm of **c** and *λ* is the regularization parameter. The problem described by (4a)–(4c) is equivalent to maximizing a convex function over a convex set. However, even for moderate values of *p*, it is computationally impractical to exhaustively enumerate all possible sets of |**c**|_0_; this problem with the *L*_0_ norm has been proven to be NP‐hard (Natarajan, 1995). Therefore, instead we consider smoothed *L*_0_ penalties: $f_{\sigma }\left(x\right)=\text{exp}\;(-\frac{x^{2}}{2^{\sigma 2}})$, for σ going to 0. In the limit, $|c{|}_{0}\approx p-\sum_{j}f_{\sigma }\;(c_{j})$, thereby the problem (4) can be approximated by

(8) $$\begin{array}{l} \max\;c^{T}G^{T}X-\lambda \left(p-\sum_{j}f\sigma (c_{j})\right)\\ c\in R^{p} \end{array},$$

subject to conditions (7a) and (7b). Equation (8) is solved for a decreasing sequence of (*σ* → 0) and a given value of *λ*, resulting in approximately sparse solutions (see Appendix C). Unfortunately there are no theoretical guarantees for the uniqueness of such numerical solutions. Often there are multiple solutions; however, when this occurs the corresponding values of the objective function (8) are usually very similar. See Appendix D for details of the solutions.

Higher values of *λ* (stronger penalization) lead to somewhat sparser solutions. In practice *λ* is chosen by K‐fold cross‐validation to minimize the projected prediction error (Kang et al., 2016) ||**P**_**G**_^∗^(**Y − X** *β* ^∗^)||, where **P**_**G**_^∗^ = **G** ^∗T^(**G** ^∗T^ **G** ^∗^)^−**1**^ **G** ^∗^ is the projector onto the columns of the genetic matrix **G** ^∗^ = **G** $\hat{c}$ given multiple solutions $\hat{c}$ . In the ideal case in which all the components of **G** ^∗^ are valid instruments (which implies the absence of pleiotropy), the regression residual **Y** − **X** *β* ^∗^ is orthogonal to the columns of **G** ^∗^: **P**_**G**_^∗^(**Y** −** X** *β* ^∗^) = 0. Notice that this orthogonality condition ensures valid solutions for constrained instrument weights, but not necessarily minimal prediction errors. More discussion about this choice of using projected prediction error as criteria for selecting the regularization parameter *λ* can be found in Appendix E.

The estimate of the exposure’s causal effect, *β*, is then obtained using approximately valid instruments **G** ^∗^. For example, in the *2SLS*, the estimator of the causal effect is given by:

$$\hat{\beta }={\left(X^{T}P_{G}⁎X\right)}^{-1}X^{T}P_{G}⁎Y.$$

Although asymptotic variance estimates are available for standard *2SLS* estimates, they are not available for the *CIV* methods. Indeed, the new instruments **G** ^∗^ depend on all observations of **X** and **Z**, so that $X_{i}G_{i}^{⁎}$ and $X_{j}G_{j}^{⁎}$ are not independent for *i* ≠ *j*. As a consequence, this weak law of large numbers cannot be invoked, and the convergence of $\frac{1}{n}\sum_{i=1}^{n}X_{i}{G^{⁎}}_{i}$ to ***E*** **|** $X_{i}{G^{⁎}}_{i}$| is not assured. Instead, bootstrap estimates of the sample variance of $\hat{\beta }$ can be obtained.

*CIV_naive* can be extended to two‐sample causal effect estimation. The weight **c** can be estimated on the first sample, and then applied to the second sample for causal effect estimation. In contrast, *CIV_smooth* can benefit from an external validation sample where (**G**, **X**, and **Y**) are all available in two data sets. These adaptations to more general study designs are included in our simulations below.

In this paper, MR analyses were restricted to the case of a single risk factor **X**, although most of the mentioned methods can be extended in some way to allow for a multivariate **X**. For *CIV_naïve* and *CIV_smooth*, we demonstrated how to account for multivariate **X** in Appendices B and C, respectively. The corresponding multiple solutions $\hat{c}$ can be used with multivariate *2SLS* to infer the causal effect of **X** on **Y**.

In summary, *CIV_naive* and *CIV_smooth* are formulated as optimization problems, which ensures that the resulting instrument **G** ^∗^ is strong and valid for estimating the causal effect of **X** on **Y**. However, in the construction algorithm for *CIV_smooth*, we cannot prove convergence to a unique solution for weight **c**, nor can we establish an analytical form for the variance of **c** and the estimate of *β*. In contrast, the most traditional benchmark in MR literature, *2SLS*, although always producing consistent estimates for *β* with an asymptotic formula for its variance, is not designed to produce strong and valid instruments. This validity concern is addressed in both the *Allele* score method and in *sisVIVE*, which can both be seen as natural competitors of our approach. Therefore, we designed and carried out a simulation study to compare *CIV_naive* and *CIV_smooth* with *Allele*_score and *sisVIVE* methods as well as the benchmark *2SLS*. These methods are available as an R package, CIVMR, at https://github.com/GreenwoodLab.

## 4 SIMULATION

The purpose of this simulation study is to assess the performance of our novel approach and of the three most popular methods over a broad variety of scenarios that mimic what we would expect to find in genetic studies. Two scenarios of pleiotropy were simulated in two series of simulations. Also, the association parameters *α*_*X*_ and *α*_*Z*_ were varied to study the impact of instrument strength on performance. Both one‐sample and two‐sample/validation sample set‐up were simulated in each of the two scenarios.

### 4.1 Simulation design

With reference to Figure 2, we capture possible violations of the MR assumptions by varying the parameters *α*_*z*_, *α*_*x*_, *γ*_*xz*_, and *γ*_*zx*_, while keeping *η* and *β* fixed, as well as the association of the unknown confounder **U** with **X**, **Z**, and **Y**. For a given set of conditions corresponding to a violation of the MR assumptions, we simulated a set of independent genotypes **G**, exposures **X**, pleiotropic phenotypes **Z**, an unknown confounder **U**, and outcome **Y**. We have implemented two series of simulations corresponding to the following scenarios:

*Series I*. Standard pleiotropy: The pleiotropic phenotype **Z** is not directly associated with **X** (*γ*_*xz*_ = *γ*_*zx*_ = 0 in Figure 2).

*Series II*. **Z** → **X**: Direct causal pathway from **Z** to **X** and **G** to **Z** (*γ*_*zx*_ ≠ 0 and *γ*_*xz*_ = 0 in Figure 2).

Table 2 summarizes the general design of the simulations. Notice that the following elements are the same in the two series: *n*, the number of observations; *p*, the total number of SNPs (components of **G**); and MAF, the minor allele frequency of each SNP. In contrast, the following parameters vary in both series: *p*_*z*_, the number of SNPs that have direct causal impact on **Z** (two values); *α*_*x*_, the association parameter between **G** and **X** (two values); *α*_*z*_, the association parameter between **G** and **Z** (two values). In total there are 2 × 2 × 2 = 8 combinations of parameters. Finally, (*γ*_*xz*_, *γ*_*zx*_) were set to (0, 0) in Series I and (0, 0.1) in Series II.

**Table 2:** The parameter settings used in the two series of simulations

Simulation	*n*	*β*	*η*	*p*	*p_z_*	MAF	(*α_x_*, *α_z_*)	*γ_xz_*	*γ_zx_*
I. Standard pleiotropy	500	1	1	100	20;50	0.33	(1,1); (1,0.1); (0.1,1); (0.1,0.1)	0	0
II. **Z** → **X**	500	1	1	100	20;50	0.33	(1,1); (1,0.1); (0.1,1); (0.1,0.1)	0.1	0

*Note*. *α_x_*: association parameter between **G** and **X**; *α_z_*: association parameter between G and Z; MAF: minor allele frequency of all SNPs in the simulation; *n*: number of individuals; *p*: number of genotypes in total; *p_z_*: number of pleiotropic genotypes with effects on **Z**.

**Structural equations for simulation Series I: Standard pleiotropy**

(9) $$\begin{array}{l} x_{i}=\alpha_{x}\sum_{j=1}^{p}G_{ij}+u_{i}+\varepsilon_{x,i},\\ z_{i}=\alpha_{z}\sum_{j=1}^{p_{z}}G_{ij}+u_{i}+\varepsilon_{z,i},\\ y_{i}=x_{i}+z_{i}+u_{i}+\varepsilon_{y,i}, \end{array}$$

where *ε_x,i_*, *ε_z,i_*, *ε_y,i_*, *u_i_*∼*N*(0, 1).

**Structural equations for simulation Series II: Z → X**

(10) $$\begin{array}{l} z_{i}=\alpha_{z}\sum_{j=1}^{p_{z}}G_{ij}+u_{i}+\varepsilon_{z,i},\\ x_{i}=\alpha_{x}\sum_{j=1}^{p}G_{ij}+\gamma_{zx}z_{i}+u_{i}+\varepsilon_{x,i},\\ y_{i}=x_{i}+z_{i}+u_{i}+\varepsilon_{y,i}, \end{array}$$

where *ε*_*x,i*_ *, ε*_*z,i*_ *, ε*_*y,i*_ *, u*_*i*_∼*N*(0, 1).

In each simulated data set, 100 SNPs (**G** in Equation (9)) with a minor allele frequency of 0.33 and *n* = 500 observations were generated, with values coded as (0, 1, 2). Among all 100 SNPs there are *p*_*z*_ ∈ {20, 50} SNPs directly related to **Z**. Notice that smaller values of *α*_*x*_ = 0.1 represent weak instruments **G** for **X**, while large values of *α*_*z*_ = 1 represent strong instruments **G** for **Z**. Therefore, our scenarios comprise weak and strong instruments for one or both of **X** and **Z**. Two hundred datasets were generated for each scenario, and results compared the estimates and variance of the causal effect, *β*.

We conducted both one‐sample and validation sample simulations for Series I and II. In one‐sample simulations we compared the bias of causal effect estimators across all the methods discussed in this study: *2SLS_naive*, *2SLS_exo*, *2SLS_mul*, *Allele*, *Allele_exo*, *Allele_mul*, *sisVIVE*, *sisVIVE_exo*, *CIV_naive*, and *CIV_smooth*. The strength of constructed instruments **G** ^∗^, and correlation between **G** ^∗^ and **Z**, are also compared across all methods except *2SLS_naive*, *2SLS_exo*, and *2SLS_mul*. The pleiotropic correlation of *sisVIVE* variants is presented as the maximum correlation between **Z** and genotypes selected by the methods.

In external validation sample and two‐sample simulations, we compared causal effect estimation bias, instrument strength, and pleiotropic correlation across all methods except *2SLS naive*, *2SLS_exo*, and *2SLS_mul*. As explained in Section 2.5, from the first sample, a vector of weights $\hat{c}$ is constructed and used to create a new instrument, **G** ^∗^ = **G** $\hat{c}$, which is then used to infer the causal effect $\hat{\beta }$ on the second sample. Notice that the vector of weights is obtained differently for different methods: for *Allele* score methods it is obtained from ordinary linear regression; for *sisVIVE* methods from a LASSO regression; and for *CIV* from the constrained optimization problem (4, 5a, and 5b).

The feature selection performances of *sisVIVE*, *sisVIVE_exo*, and *CIV_smooth* are also reported for all the simulation scenarios. The feature selection result from *CIV_smooth* is extracted as follows: we first obtain *CIV_smooth* estimates $\hat{c}$. For each converged solution $\hat{c}$, a feature *j* is recognized as significant if coefficient |*c*_*j*_| ≥ 0.2 × max_*j*_ |*c*_*j*_|*, j* = 1*, …, p*. All selected features are then recognized as selected valid instruments.

### 4.2 Simulation results

The simulations were designed to assess the performances of *CIV_naive* and *CIV_smooth*. The expectation was that both approaches would provide strong instruments with near zero pleiotropic correlation compared with other methods. Moreover, *CIV_smooth* should reduce the number of selected pleiotropic genotypes, thus providing more valid instruments and more accurate $\hat{\beta }$ compared with some competitors; specifically, the validity of the instruments obtained from *CIV_smooth* should be comparable with those obtained from *sisVIVE*.

#### 4.2.1 One sample simulation

**Figure 4:**
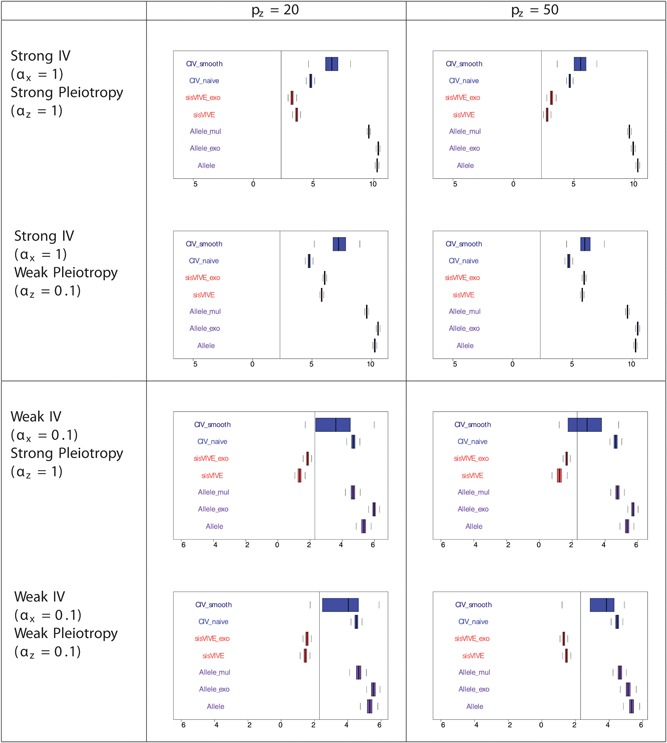
Log‐transformed *F*‐statistics of **X**~**G** ^∗^ for each Mendelian randomization method in one‐sample set‐up for simulation series I. The panels display results for different values of *α_x_* and *α_z_* corresponding to different instrument strength and pleiotropy severity. *p_z_* denotes the number of pleiotropic components among all 100 single nucleotide polymorphisms in **G**. Vertical line denotes *F*‐statistics = 10

**Figure 5:**
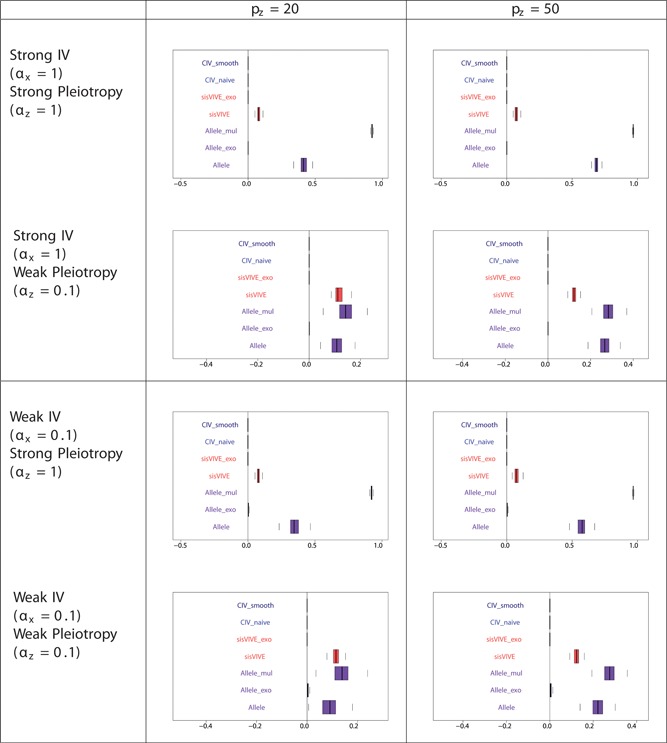
Pleiotropic correlations of **Z** and **G** ^∗^ for each Mendelian randomization method in a one‐sample set‐up for simulation Series I. The panels display results for different values of *α_x_* and *α_z_* corresponding to different instrument strength and pleiotropy severity. *p_z_* denotes the number of pleiotropic components among all 100 single nucleotide polymorphisms in **G**. Note that the pleiotropic correlations from *CIV_smooth*, *CIV_naive*, *sisVIVE_exo*, and *Allele_exo* are exactly zero in some scenarios

**Figure 6:**
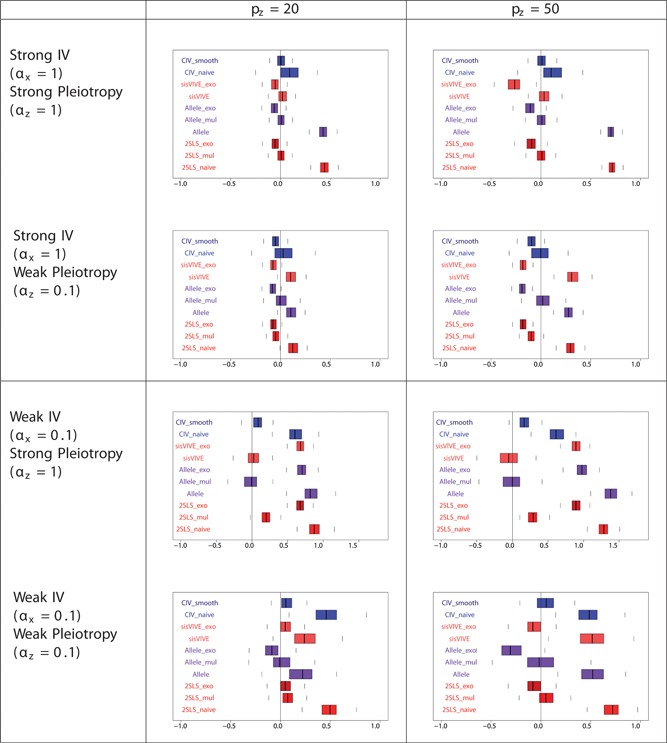
Boxplots of the bias of the causal effect estimates, β−1, from a one‐sample set‐up in simulation Series I. The panels display results for different values of *α_x_* and *α_z_* corresponding to different instrument strength and pleiotropy severity. *p_z_* denotes the number of pleiotropic components among all 100 single nucleotide polymorphisms in **G**

These expectations were met in one‐sample simulations, as shown in Figures 4, 5, 6 and Table 3 for Series I. The *F*‐statistics of Figure 4 show that the instrument strengths of *CIV_naive* and *CIV_smooth* are superior to that of *sisVIVE* and *sisVIVE_exo* across scenarios. Although, as expected, the largest instrument strengths are obtained from the three variants of the *Allele* score method; across all scenarios the *F*‐statistics of the *CIV* approaches are >10, indicating that instrument strength is retained despite the adjustments for pleiotropy. The pleiotropic correlations for one‐sample simulations, presented in Figure 5, show that, confirming our expectations, both *CIV_smooth* and *CIV_naive* have exactly zero pleiotropic correlations in all scenarios, whereas *sisVIVE*, *Allele*, and *Allele_mul* show substantial nonzero values.

**Table 3:** Feature selection results for *CIV_smooth*, *sisVIVE* and *sisVIVE_exo* from a one‐sample set‐up in simulation Series I

Scenario	Method	$p_{z}\;=\;20$		$p_{z}\;=\;50$	
		TP	FP	TP	FP
Strong IV ($\alpha_{x}\;=\;1$)	*CIV_smooth*	79.55	7.42	49.98	13.15
Strong pleiotropy ($\alpha_{z}\;=\;1)$	*sisVIVE*	73.78	0.02	36.99	0.08
	*sisVIVE_exo*	41.11	0.02	8.18	0.53
Strong IV ($\alpha_{x}\;=\;1$)	*CIV_smooth*	77.00	14.53	46.99	27.18
Weak pleiotropy ($\alpha_{z}\;=\;0.1)$	*sisVIVE*	80.00	19.98	50.00	50.00
	*sisVIVE_exo*	80.00	20.00	50.00	50.00
Weak IV ($\alpha_{x}\;=\;0.1$)	*CIV_smooth*	70.47	11.76	45.07	31.17
Strong pleiotropy ($\alpha_{z}\;=\;1)$	*sisVIVE*	68.22	0.00	32.72	0.26
	*sisVIVE_exo*	79.24	19.76	50.00	50.00
Weak IV ($\alpha_{x}\;=\;0.1$)	*CIV_smooth*	69.72	11.80	43.29	27.53
Weak pleiotropy ($\alpha_{z}\;=\;0.1)$	*sisVIVE*	80.00	20.00	49.97	49.95
	*sisVIVE_exo*	80.00	20.00	49.90	49.73

*Notes*. The panels display results for different values of *α_x_*and *α_z_*corresponding to different instrument strength and pleiotropy severity. FP: average number of selected false positive variables out of *p_z_*; *p_z_*: the number of pleiotropic components among all 100 single nucleotide polymorphisms in **G**. TP: average number of selected true‐positive variables out of 100−*p_z_*.

The feature selection results in Table 3 show that *sisVIVE* outperforms *CIV_smooth* in the strong pleiotropy case, with a smaller true positive rate and a much smaller false positive rate. In contrast, in the weak pleiotropy case, it is the *CIV_smooth* that outperforms *sisVIVE*: here the *sisVIVE* approach does not eliminate any genetic variants, whereas *CIV_smooth* correctly eliminates 30–50% of the invalid genotypes.

The bias of $\hat{\beta }$ across methods from simulation Series I in one‐sample set‐up is presented in Figure 6. For all methods, this bias is smaller for strong instrument scenarios than for weak instrument scenarios, and is higher for strong pleiotropy scenarios than for weak pleiotropy scenarios. Moreover, for all methods, the most biased estimates are those obtained from weak instruments and strong pleiotropy scenarios. It should be noted that in the latter case, $\hat{\beta }$ is unbiased for *CIV_smooth*, *sisVIVE* and *Allele_mul*. Also, *CIV_smooth* outperforms *CIV_naive* in each scenario in terms of magnitude of the $\hat{\beta }$ bias. The reason for the discrepancy between *CIV_smooth* and *CIV_naïve* is that *CIV_smooth* includes a prediction optimization procedure, whereas *CIV_naive* does not (see Appendix E).

**Figure 7:**
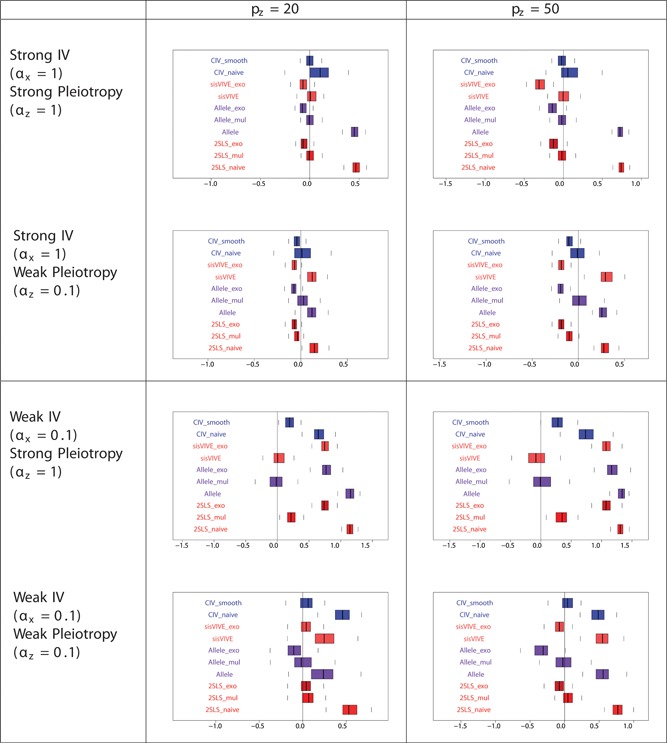
Boxplots of the bias of the causal effect estimates, β−1, from a one‐sample set‐up in simulation Series II. The panels display results for different values of *α_x_* and *α_z_* corresponding to different instrument strength and pleiotropy severity. *p_z_* denotes the number of pleiotropic components among all 100 single nucleotide polymorphisms in **G**

**Figure 8:**
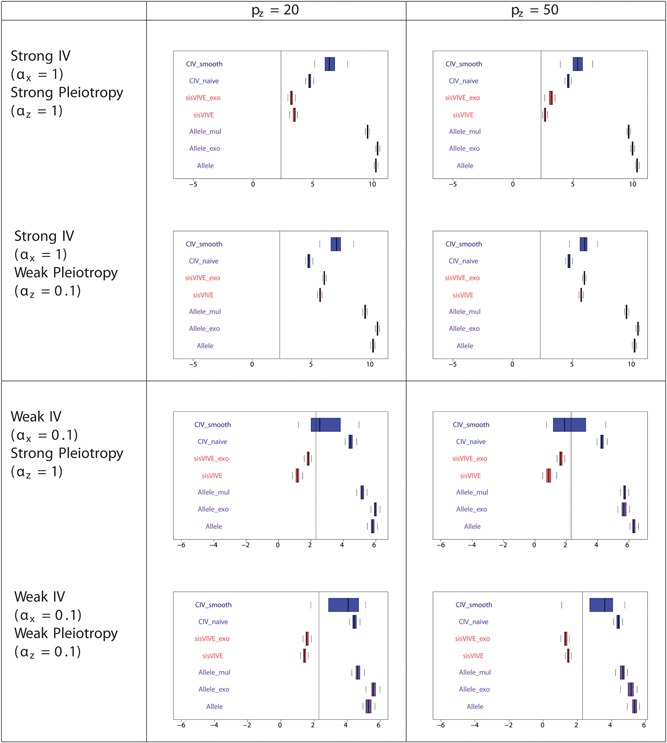
Log‐transformed *F*‐statistics of **X**~**G** ^∗^ for each Mendelian randomization method in a one sample set‐up for simulation Series II. The panels display results for different values of *α_x_* and corresponding to different instrument strength and pleiotropy severity. *p_z_* denotes the number of pleiotropic components among all 100 single nucleotide polymorphisms in **G**

**Figure 9:**
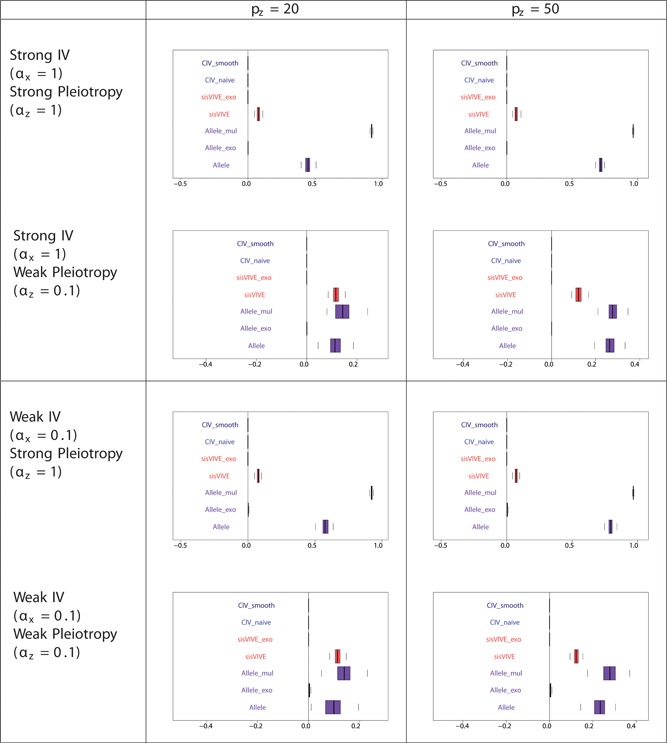
Pleiotropic correlations of **Z** and **G** ^∗^ for each Mendelian randomization method in a one sample set‐up for simulation series II. The panels display results for different values of *α_x_* and *α_z_* corresponding to different instrument strength and pleiotropy severity. *p_z_* denotes the number of pleiotropic components among all 100 single nucleotide polymorphisms in **G**. Note that the pleiotropic correlations from *CIV_smooth*, *CIV_naive*, *sisVIVE_exo*, and *Allele_exo* are exactly zero in some scenarios, and, therefore, do not appear on the graphs

The simulation results for Series II in the one sample set‐up, in general, are similar to those for Series I (Figures 7, 8, 9, and Table 4 for Series II). The only difference between the results of Series II and I lies in the performance of *2SLS_mul*: unlike Series I, the estimates of $\hat{\beta }$ from *2SLS_mul* are significantly biased in the scenario of weak instruments and strong pleiotropy from Series II (Figure 7). When both pleiotropy and instruments are strong (first row of Figure 7) methods conditional on **Z** (*sisVIVE_exo*, *Allele_exo*, and *2SLS exo*) give smaller estimates of $\hat{\beta }$ than those without conditioning on **Z** (*sisVIVE*, *Allele*, and *2SLS*). This is the collider bias induced by conditioning on **Z**. The same pattern can be seen in two other rows of Figure 7. A different pattern is seen when the instruments are weak but the pleiotropy is strong (third row of Figure 7). Conditioning on **Z** in this situation may be exacerbating the imprecision due to weak instruments.

**Table 4:** Feature selection results for *CIV_smooth*, *sisVIVE,* and *sisVIVE_exo* from a one‐sample set‐up in simulation series II

Scenario	Method	*p_z_* = 20		*p_z_* = 50	
		TP	FP	TP	FP
Strong IV ($\alpha_{x}\;=\;1$)	*CIV_smooth*	79.60	6.86	48.94	12.29
Strong pleiotropy ($\alpha_{z}\;=\;1)$	*sisVIVE*	73.23	0.00	36.39	0.04
	*sisVIVE_exo*	39.85	0.00	8.34	0.52
Strong IV ($\alpha_{x}\;=\;1$)	*CIV_smooth*	77.19	15.06	47.20	26.93
Weak pleiotropy ($\alpha_{z}\;=\;0.1)$	*sisVIVE*	80.00	20.00	50.00	49.97
	*sisVIVE_exo*	80.00	20.00	59.98	49.98
Weak IV ($\alpha_{x}\;=\;0.1$)	*CIV_smooth*	70.68	12.09	46.48	34.62
Strong pleiotropy ($\alpha_{z}\;=\;1)$	*sisVIVE*	68.54	0.00	31.92	0.04
	*sisVIVE_exo*	78.48	19.52	50.00	50.00
Weak IV ($\alpha_{x}\;=\;0.1$)	*CIV_smooth*	70.06	12.03	43.75	27.48
Weak pleiotropy ($\alpha_{z}\;=\;0.1)$	*sisVIVE*	80.00	20.00	49.98	49.93
	*sisVIVE_exo*	79.97	19.93	50.00	50.00

*Notes*. The panels display results for different values of *α_x_* and *α_z_* corresponding to different instrument strength and pleiotropy severity. FP: average number of selected false positive variables out of *p_z_*; *p_z_*: the number of pleiotropic components among all 100 single nucleotide polymorphisms in G. TP: average number of selected true positive variables out of 100−*p_z_*.

#### 4.2.2 Two sample simulation

**Figure 10:**
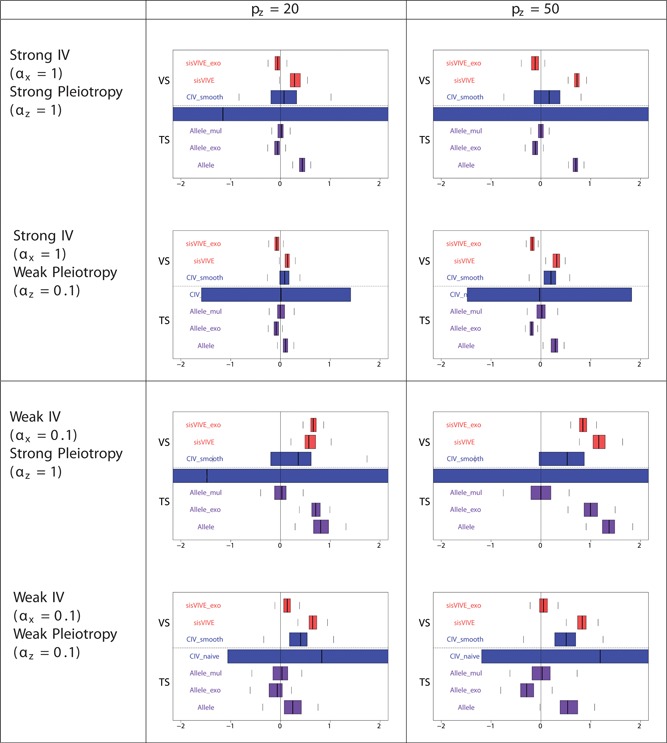
Boxplots of the bias of the causal effect estimates, β−1, from external validation sample and two‐sample set‐ups in simulation Series I. The panels display results for different values of *α_x_* and *α_z_* corresponding to different instrument strength and pleiotropy severity. *p_z_* denotes the number of pleiotropic components among all 100 single nucleotide polymorphisms in **G**. The dashed line separates external VS results from TS results. TS: two‐sample; VS: validation sample

Two‐sample and external validation sample bias results are shown in Figure 10 for Series I simulations. External validation sample results are shown above the horizontal line in each image, and two‐sample results are shown below the line, only for methods that adapt to these situations.

**Figure 11:**
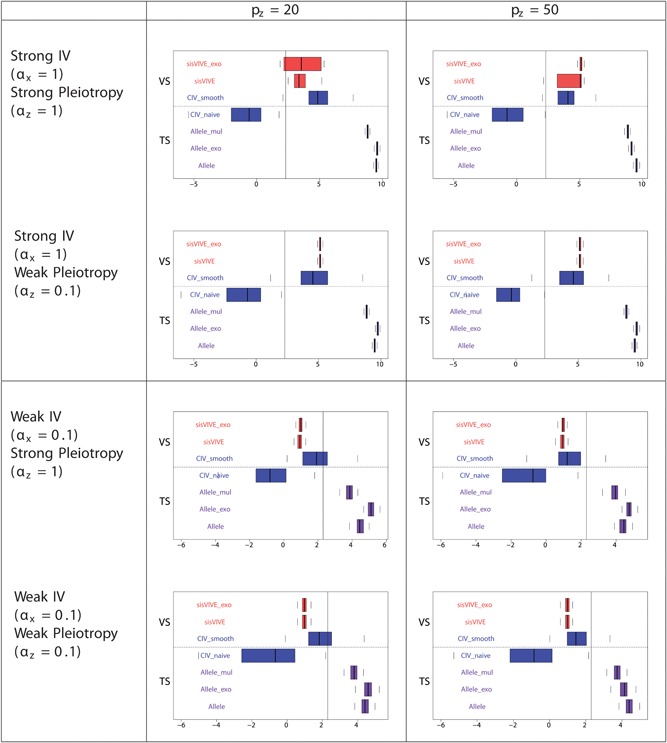
Log‐transformed *F*‐statistics of **X**~**G** ^∗^ for each Mendelian randomization method (in the second sample) in simulation Series I. The panels display results for different values of *α_x_* and *α_z_* corresponding to different instrument strength and pleiotropy severity. *p_z_* denotes the number of pleiotropic components among all 100 single nucleotide polymorphisms in **G**. Vertical line denotes *F*‐statistics = 10. The dashed line separates external VS results from TS results. TS: two‐sample; VS: validation sample

**Figure 12:**
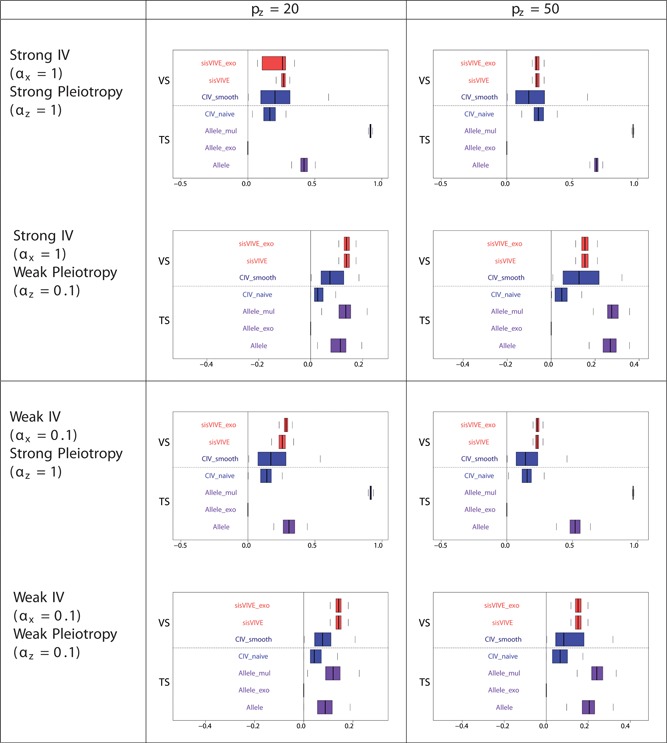
Pleiotropic correlations of Z and G^∗^ for each Mendelian randomization method (in the second sample) in simulation Series I. The panels display results for different values of *α_x_* and *α_z_* corresponding to different instrument strength and pleiotropy severity. *p_z_* denotes the number of pleiotropic components among all 100 single nucleotide polymorphisms in **G**. Note that the pleiotropic correlation values from *Allele_exo* are exactly zero in some scenarios. The dashed line separates external VS results from TS results. TS: two‐sample; VS: validation sample

Across all scenarios, *CIV_naive* has substantially larger variances than all other methods, to the extent that it is impossible to even evaluate bias. *CIV_naive* is not designed to optimize prediction of **Y** in the second sample, and does not use **Y** in the instrument construction process of the first sample. Hence, *CIV_naive* weights are not robust across data sets. In fact, the instrument strengths for *CIV_naive* in two‐sample set‐up (Figure 11) are significantly lower than in one‐sample set‐up (Figure 4). Figure 12 shows that the pleiotropic correlations of *CIV_naive* from the two‐sample set‐up are larger than those from the one‐sample simulations (Figure 5), for the same reason.

For the two‐sample set‐up, *Allele_mul* gives unbiased results in all scenarios; however, the *Allele_exo* is biased in the scenario of weak instruments and strong pleiotropy. In general, across all scenarios, the strongest instruments as well as highest pleiotropic correlations occur for the three variants of the *Allele* score method.

**Figure 13:**
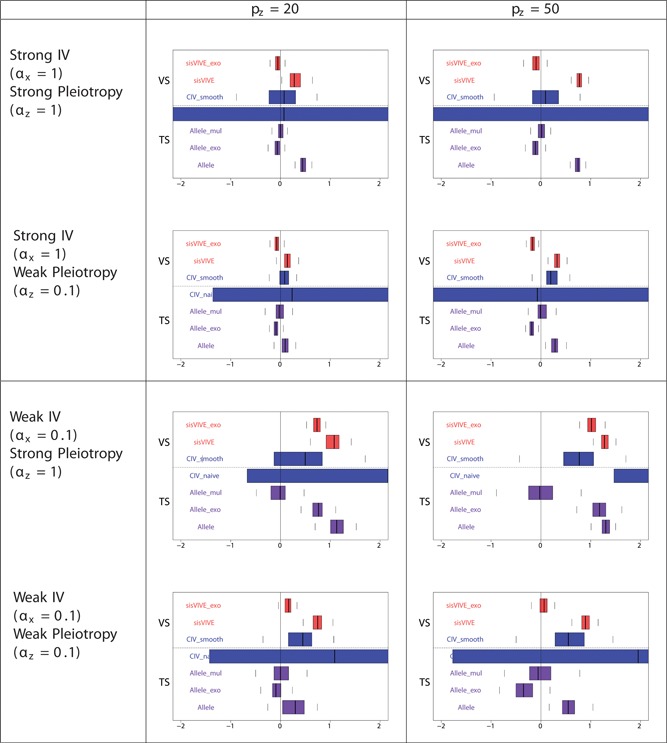
Boxplots of the bias of the causal effect estimates, β‐1, from external validation sample and two‐sample set‐ups in simulation Series II. The panels display results for different values of *α_x_* and *α_z_* corresponding to different instrument strength and pleiotropy severity. *p_z_* denotes the number of pleiotropic components among all 100 single nucleotide polymorphisms in **G**. The dashed line separates external VS results from TS results. TS: two‐sample; VS: validation sample

**Figure 14:**
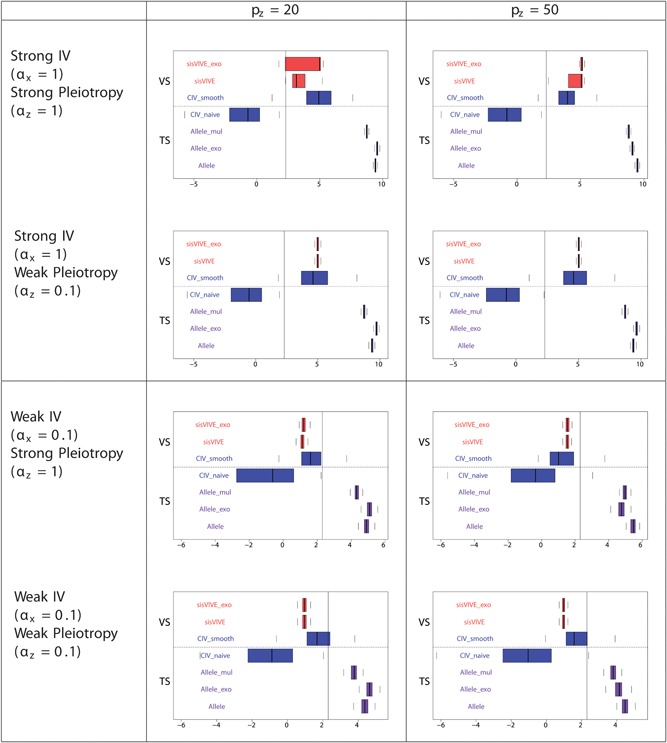
Log‐transformed *F*‐statistics of **X**~**G** ^∗^ for each Mendelian randomization method (in the second sample) in simulation Series II. The panels display results for different values of *α_x_* and *α_z_* corresponding to different instrument strength and pleiotropy severity. *p_z_* denotes the number of pleiotropic components among all 100 single nucleotide polymorphisms in **G**. Vertical line denotes *F*‐statistics = 10. The dashed line separates external VS results from TS results. TS: two‐sample; VS: validation sample

**Figure 15:**
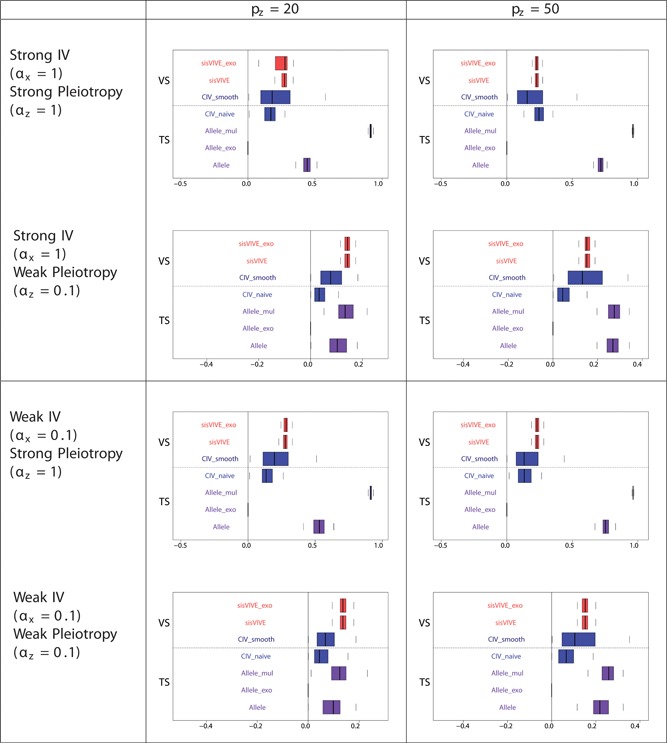
Pleiotropic correlations of **Z** and **G** ^∗^ for each Mendelian randomization method (in the second sample) in simulation series II. The panels display results for different values of *α_x_* and *α_z_* corresponding to different instrument strength and pleiotropy severity. *p_z_* denotes the number of pleiotropic components among all 100 single nucleotide polymorphisms in **G**. Note that the pleiotropic correlation values from *Allele_exo* are exactly zero in some scenarios, and therefore nothing can be seen on the graphs. The dashed line separates external VS results from TS results. TS: two‐sample; VS: validation sample

The simulation results for Series II in two‐sample set‐up are similar to those for Series I: they are shown in Figures 13, 14, 15.

#### 4.2.3 External validation sample simulation

The validation sample simulation results confirm our hypothesis that *CIV_smooth* is more robust than *CIV_naive*; *CIV_smooth* is unbiased in all scenarios and is much less variable than *CIV_naive*. It is likely that *CIV_smooth* attains robustness by incorporating a penalty approach to select genotypes, and uses **Y** in the instrument construction process to optimize projected predictions of **X** on **Y**, thus achieving greater stability of $\hat{\beta }$.

The *F*‐statistics of *CIV_smooth* from Series I in the external validation‐sample set‐up (Figure 11) are substantially lower than the *F*‐statistics from the one‐sample set‐up, as might be expected. Also, the pleiotropic correlations of *CIV_smooth* from the two‐sample set‐up (Figure 12) are larger than those from the one‐sample simulations (see Figure 5) for the same reason as was seen for *CIV_naive* above. In general, across all scenarios, the estimated instrument strength and the pleiotropic correlation of *CIV_smooth* are comparable with those of *sisVIVE* and *sisVIVE_exo*.

### 4.3 Simulation summary

In conclusion, *CIV_smooth* and *Allele_mul* methods provide the only unbiased causal effect estimates in all scenarios. The *sisVIVE* estimates are biased in some scenarios, especially for weak pleiotropy scenarios. *Allele_mul* retains high pleiotropic correlation when strong pleiotropy exists, as it does not select the components of **G**. The estimated instrument strength and pleiotropic correlation of *CIV_smooth* are always close to those of its close competitors; moreover they are the only unbiased casual effect estimation method that performs feature selection.

## 5 DATA ANALYSIS: THE ALZHEIMER’S DISEASE NEUROIMAGING INITIATIVE (ADNI) COHORT

AD is a chronic neurodegenerative disorder that causes a slow decline in memory and reasoning skills. It is well known that biomarkers, including cerebrospinal fluid tau protein (CSF‐tau) and cerebrospinal fluid Aβ‐protein ending at amino acid position 42 (CSF‐Aβ 1–42), are reliable measures of AD progression (Frost, Jacks, & Diamond, 2009; Hardy & Higgins, 1992; Shaw et al., 2009). Recently, other biomarkers such as flluoro‐D‐glucose standardized uptake (FDG_SUVR) and neural functional activity have been added when exploring the mechanisms underlying late‐onset Alzheimer’s disease (LOAD) using multifactorial data analysis (Iturria‐Medina, Sotero, Toussaint, Mateos‐Pérez, & ADNI, 2016). However, at this point, there is still uncertainty as to whether the changes in these biomarkers play a causal role in AD progression or are simply associated with AD progression.

We have used instrumental variable methods to try to disentangle causal relationships for AD. Data used in the preparation of this study were obtained from the ADNI database (adni.loni.usc.edu). The ADNI study was launched in 2003 as a public–private partnership, led by Principal Investigator Michael W. Weiner, MD. The primary goal of ADNI has been to test whether serial magnetic resonance imaging, positron emission tomography, other biological markers, and clinical and neuropsychological assessment can be combined to measure the progression of mild cognitive impairment (MCI) and early AD.

The outcome (AD status) studied here is a binary case–control variable, where “case” is a subject with either MCI or early AD. Thus we use logistic regression in the second stage of MR analysis to estimate a causal risk ratio (CRR; Burgess, Granell, Palmer, Sterne, & Didelez, 2014; Clarke & Windmeijer, 2012). The *sisVIVE* method, however, requires the outcome to be continuous; therefore, we adjusted outcome **Y**, **X**, and **Z** for the exogenous covariates sex, education, and age, and replaced these with their predictors $\hat{Y}$ (which can be considered quasicontinuous), $\hat{X}$ and $\hat{Z}$, to which we can apply *sisVIVE*. In this case, any bias toward the null in the causal effect estimates from *sisVIVE* would be largely due to the impact of confounding factors (Palmer, Thompson, Tobin, Sheehan, & Burton, 2008).

A very important limitation of performing MR analysis in ADNI data is the retrospective nature of its study design. Ascertainment in ADNI was retrospective by disease status, and therefore, instruments that would be valid for a prospective study design may not remain valid after retrospective sampling (Didelez & Sheehan, 2007). Specifically, the estimated first stage (**X**∼**G**) association from case–control samples may be biased relative to the true association in a general population sample (Tapsoba, Kooperberg, Reiner, Wang, & Dai, 2014; Tchetgen Tchetgen, 2013). If the disease being studied is rare, it is possible to conduct a first stage regression only on the control sample, then perform causal effect estimation on the whole sample using MR methods applicable to two‐sample/validation sample set‐ups (Lin & Zeng, 2009).

For illustration of *CIV* below, we select in turn each of the four available biomarkers (CSF‐Aβ 1–42, CSF‐Ptau, CSF‐Ttau and FDG_SUVR) as **X**, and then assign the other three to be the pleiotropic phenotypes, **Z**. This then raises another limitation of our MR analysis of these data: We are assuming there is no causal relationship from **X** → **Z** as this would imply a different total causal effect than the one that we are estimating. Given our rotation of phenotypes between the (**X**, **Z**) position, we are essentially assuming there is no direct causal relationship between any of these phenotypes and that pleiotropy is induced merely by sharing some genetic contributions. Therefore, we suggest that the results below should be interpreted as simply illustrating our methods and not as making substantive causal statements.

### 5.1 Outcome, exposures and instruments

***Outcome*** **Y:** A subject is either from the control group, or is a “case” if diagnosed with MCI or AD. In total we analyzed *n* = 491 subjects including 151 controls (**Y** = 0) and 340 cases (**Y** = 1).

***Exposures*** **X:** We are interested in estimating the causal effect on AD progression of four biomarkers, including CSF‐Aβ 1–42 (*X*_1_), natural log of Ptau (*X*_2_), natural log of Ttau (*X*_3_) and FDG_SUVR (*X*_4_). It is well known that the isoforms of apolipoprotein E, a class of apolipoprotein that mediates cholesterol metabolism, are associated with both Aβ aggregation and Tau protein phosphorylation (Brecht et al., 2004; Frautschy & Cole, 2010; Strittmatter & Roses, 1995; Sunderland et al., 2004), which implies potential pleiotropy. If there were multiple measurements of the biomarkers, the first one was used. All exposure variables were adjusted for covariates age, sex, and education. Profiles of the subjects are summarized in Table 5.

**Table 5:** Characteristics of subjects studied in ADNI

	Number	Age (years; mean ± *SD*)	Gender (M/F)	Education (years; mean ± *SD*)
Control	151	75.93 ± 5.86	86/65	16.3 + 2.7
MCI/AD	340	74.08 ± 7.63	212/128	15.89 ± 2.92
MCI	277	73.64 ± 7.53	173/104	16.03 ± 2.81
AD	63	76.03 ± 7.78	39/24	15.27 ± 3.31

*Note*. AD: Alzheimer’s disease; ADNI: Alzheimer’s disease neuroimaging initiative; MCI: mild cognitive impairment.

***Instruments*** **G:** For each of the exposures *X*_*k*_ *, k* = 1, *…*, 4, the strongly associated SNPs reported by the NHGRI‐EBI catalog of published genome‐wide association studies (Burdett et al., 2016) were collected from the ADNI Imputed Genotype data. The missing genotypes were imputed based on the 1,000 Genome Project, utilizing the same protocol for the ROS/MAP and AddNeuroMed study. When there were very highly correlated (*ρ* ≥ 0.8) SNPs which are known to belong to the same gene, we kept only one representative SNP. The SNP set was then further reduced by using a univariate feature selection based on significant *F*‐statistics (*p* ≤ 0.05). Hence, the final selected SNPs comprised 12 SNPs for Aβ (*X*_1_), six SNPs for Ptau (*X*_2_), four SNPs for Ttau (*X*_3_), and 17 SNPs for FDG_SUVR (*X*_4_).

### 5.2 MR analysis

**Figure 16:**
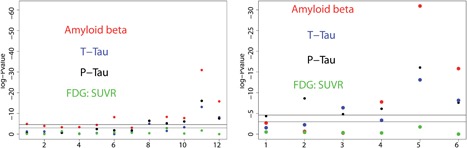
Strength of association, measured by ‐log10 pvalues, between all four biomarkers and SNPs selected through their asssociation with one biomarker. Left: SNPs selected for Amyloid beta; Right: SNPs selected for Ptau

**Figure 17:**
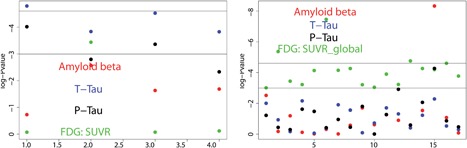
Strength of association, measured by ‐log10 pvalues, between all four biomarkers and SNPs selected through their asssociation with one of the biomarkers. Left: SNPs selected for Ttau; Right: SNPS selected for SUVR.

The assumption (A1) of MR states that the SNPs must be associated with biomarkers of interest. Strong instruments with *F*‐statistics bigger than 10 are usually preferred in MR applications. The *F*‐statistics for instrument strength, based on the set of SNPs selected for each biomarker, were 12.44 (Aβ), 12.01 (Ptau), 4.52 (Ttau), and 5.94 (FDG_SUVR). We also performed the Sargan test for over‐identification (Baum et al., 2003) to test the MR assumption (A2) and (A3). The *p* values of the Sargan test were 1.5e−4, 5e−5, 0.23, and 3e−4 for *X*_*k*_, *k* = 1, *…*, 4, implying the existence of invalid instruments in **G** for MR for Aβ (*X*_1_), Ptau (*X*_2_), and FDG_SUVR (*X*_4_) on AD progression (**Y**). The reason for these small *p*‐values is that the selected SNPs that are strongly associated with Ptau have even stronger associations with Aβ. Individual p‐values for instruments **G** with the four biomarkers are shown in Figures 16 and 17.

MR was performed to evaluate the potential causal effects of variability in each biomarker (**X**) on the AD progression (**Y**) in two steps. In the first step, we used only the control samples to obtain weights with applicable methods (*Allele* methods, *sisVIVE* methods and *CIV_smooth*). In the second step, using the whole sample, we constructed instrumental variables using the weights obtained from the first step, and inferred causal effects of each biomarker *X*_*k*_ on AD progression while adjusting for the other three biomarkers as secondary phenotypes. In this set‐up, if we assume that the control sample is similar to the whole population from which the individuals were drawn, then the retrospective nature of ADNI is respected. As said above, we also acknowledge that our analyses assume no causal relationship from **X** to **Z** for each (**G**, **X**, **Z**, **Y**) set‐up, and results need to be interpreted in this light. It is important to note that in this analysis, we can only include *CIV_smooth*, *Allele* scores, and *sisVIVE* because not all methods can be adapted to this two‐step approach. We excluded *CIV_naive* due to its unstable performance in the two‐step approach (see Section 4.2).

### 5.3 Results

**Figure 18:**
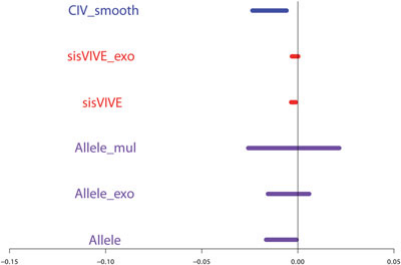
95% bootstrapped confidence interval of causal estimates (log odds) of CSF‐A β 1–42 protein levels on AD progression using *CIV_smooth* and *Allele* methods in external validation sample set‐ups. It is important to note that the confidence interval shown here for *sisVIVE* methods results from treating **Y** as continuous, since *sisVIVE* is not designed for binary outcomes. These results show a decrease in AD risk with higher amyloid beta levels.

Using *CIV_smooth* we found a significant causal effect of CSF‐Aβ 1–42 on AD progression, with lower CSF‐Aβ 1–42 levels in AD patients than controls. The 95% confidence intervals of the causal effect estimates (log‐odds) for CSF‐Aβ 1–42 obtained from two‐sample/validation sample analyses are reported in Figure 18. Neither the three variants of *Allele* score methods, nor the two variants of *sisVIVE* methods identified a significant causal effect of CSF‐Aβ 1–42 peptide levels on AD progression. In contrast, none of the methods found significant causal effects for Ttau, Ptau, and FDG_SUVR on AD progression.

The observation of a significant causal impact for CSF‐Aβ 1–42 on AD is consistent with some previous publications. In fact, multiple observational studies have reported decreasing Aβ 1–42 in cerebrospinal fluid of patients with AD compared with normal control subjects (Herukka et al., 2007; Maruyama et al., 2001; Sunderland et al., 2003). However, as mentioned above, these results are merely illustrative of the performance of our methods.

## 6 DISCUSSION

In this paper we proposed a new *CIV* method for causal inference when pleiotropy is suspected. This method, *CIV_smooth*, is an improved variant of a conceptually simpler one, *CIV_naive*, which is defined within the broader framework of instrumental variable theory. *CIV_naive* optimizes an objective function under a “hard” constraint; *CIV_smooth* adds to this a soft constraint to favor smoothed *L*_0_ solutions. In our simulation study, we have presented and compared the performance of *CIV_smooth*, *CIV_naive*, and other popular methods. A variety of simulation scenarios were constructed to mimic realistic pleiotropic relationships. We found that *CIV_smooth* compares favorably to its closest competitors with respect to instrument strength, pleiotropic correlation, and causal effect estimation bias in a one‐sample analysis design. We note furthermore that *CIV_naive*, while outperforming its competitors in specific situations, is uniformly outperformed by *CIV_smooth*. To illustrate the performance of *CIV_smooth* and its competitors, we conducted MR analysis on data from ADNI (Mueller et al., 2005), with the aim of estimating the causal effects of the biomarkers CSF‐Aβ 1–42, CSF‐Ptau, CSF‐Ttau and FDG_SUVR on AD progression. *CIV_smooth* found only one significant causal effect, that of CSF‐Aβ 1–42 on AD progression; this suggests that the previously known association of this biomarker with AD progression may be causal. In contrast, all the other methods failed to uncover any significant causal effect.

The main advantage of the *CIV_smooth* method is that it constructs valid instruments that are strongly associated with a phenotype of interest. Indeed by construction, *CIV_smooth* aims to balance the “validity” (pleiotropic correlation) and instrument strength (association with phenotype) of solutions. This balance is desirable, since strong instruments will provide consistent causal effect estimates, whereas approximately valid instruments will reduce the pleiotropy‐induced bias. The simulations show that *CIV_smooth* provides unbiased causal effect estimates by achieving this balance; although it could be slightly outperformed by its competitors on either pleiotropic correlation or instrument strength, but not both. At the same time, in one‐sample analyses the novel feature selection aspect of *CIV_smooth* does not introduce significant bias in causal effect estimation.

Another advantage of *CIV_smooth* is the option of separating instrument construction and causal effect estimation. In fact, the construction of *CIV_smooth* instruments relies on a coefficient vector **c** estimated from a sample of **G**, **X**, **Z**, **Y**. Then, any estimation method for linear structural equations can be applied to *CIV_smooth* instruments **G** ^∗^ → **X → Y** for causal inference. Due to this separation of first‐stage and second‐stage analysis, *CIV_naive*, *CIV_smooth*, and *Allele* scores can be trained and assessed on different datasets. It should be noted that the consistency of *CIV_smooth* is reasonable and comparable with that of its closest competitors, while *CIV_naive* is often found to be severely inconsistent. Therefore, it is clear that *CIV_smooth* has substantial flexibility in terms of model assessment and causal effect estimation.

In the presence of pleiotropic phenotypes **Z** (*α*_*z*_ ≠ 0 in Figure 2), any method that conditions on **Z** would induce collider bias. The main advantage of *CIV_smooth* is to propose a selection of valid instruments **G** ^∗^ that are meant to approach, as closely as possible, the ideal situation, *α*_*z*_^∗^ = 0. Nevertheless, collider bias in *CIV_smooth* will still be induced when the pleiotropic correlation between **G** ^∗^ and **Z** is high in absolute values. However, Simulation Series I and II show that *CIV_smooth* is more robust than *sisVIVE* and *2SLS* methods even though spurious association may have been introduced by the constrained projections (see Figures 7, 8, 9 and 13, 14, 15). We plan additional investigation in future work.

In this paper we did not consider scenario (iii) of Section 2.1 corresponding to *γ*_*xz*_ ≠ 0, in which the total causal effect of **X** on **Y** includes a contribution through **Z**. In this scenario, we do have a true **X → Z** relationship with total causal effect of *β* + *γ_xz_* *η*. Therefore *sisVIVE* is unlikely to perform well if there are pleiotropic genotypes since by its definition all genotypes are invalid for **X** (a valid genotype only impacts **Y** through **X**). Also, neither *CIV_naive* nor *CIV_smooth* estimate *β + γ_xz_η*, and generalizations do not seem to be easy. Although some preliminary simulation results including an **X → Z** causal relationship did show that *CIV_smooth* may have potential in this scenario, in that the direct causal effect *β* of **X** on **Y** can be estimated adequately, a good estimate of the total causal effect requires extensive changes to the present methodology, and is beyond the scope of this paper. Leaving out scenario (iii) is certainly a limitation of this study that will require further careful research. On the other hand, even with this limitation the results of this paper have practical applications. Indeed, it may be plausible that any correlation between **Z** and **X** is due to a “common cause” and not to any causal relationship **X → Z** or **Z → X**, in which case this “common cause” would be absorbed by **U** and fall under the case we consider here (both *γ*_*xz*_ and *γ*_*zx*_ are zero). We note further that conditioning on **Z** when *γ*_*xz*_ ≠ 0 may exacerbate collider bias.

A major limitation of our method is the multiplicity of solutions occurring in certain regions of the parameter space. We have attempted to alleviate this problem by launching the algorithm from multiple initial points, and combining the resulting instruments into a matrix (observation × instrument), which becomes itself an instrument **G** ^∗^ (see Appendix C for details).

Another limitation of *CIV_smooth* is the ad hoc choice of the threshold used in the variable selection step. In this study we are fixing the threshold at 0.2, an empirical choice based on our simulation (see Section 4.1 for details). However, this choice may be problematic in applications featuring large numbers of pleiotropic genotypes.

A third limitation of *CIV_smooth* is its failure to eliminate the influence of pleiotropic phenotypes when **Z** contains only some but not all pleiotropic phenotypes. We conducted a sensitivity analysis of *CIV_smooth*, varying the proportion of observed pleiotropic phenotypes. The results show that in most scenarios *sisVIVE* and *sisVIVE_exo* methods estimate the causal effects with the smallest bias among all competitors. However, if *α*_*z*_ is small and more than 50% of pleiotropic phenotypes are observed (in **Z**), then *CIV_smooth* does provide better (smaller bias) causal effect estimates than *sisVIVE* methods and *Allele* methods. This result points to some avenues for future research through investigations of robustness to improve the performance of *CIV_naive* and *CIV_smooth*. See Appendix F for details (particularly Supporting Information Figures S3 and S5).

In our MR analysis of the ADNI data set, an important limitation is that ADNI is a retrospectively designed study. In an attempt to alleviate this problem, we implemented the two‐stage approach, introduced by Jiang, Scott, and Wild (2006): in the first stage weighted scores were constructed from the control samples and in the second‐stage instruments were constructed with these scores on the whole data set, and the causal effect of each individual biomarker was estimated while treating the other biomarkers as secondary phenotypes. However, this two‐stage approach cannot completely resolve the problems associated with using an MR approach on a retrospective study (Bowden & Vansteelandt, 2011; Tchetgen Tchetgen, Walter, & Glymour, 2013).

Another limitation of the ADNI data analysis is that we treated causal effect estimation for multiple phenotypes as a series of estimations, each with one of the phenotypes as **X** and the others as **Z**. This reduction was necessary to compare methods, since only *CIV* allows multivariate versions of both **X** and **Z**. However, such set‐up is only appropriate when there is no direct causal impact **X → Z** for each pair (**X**, **Z**), in which case the total causal effect of **X** on **Y** is equal to the direct causal effect. If this assumption is not true for any pair of (**X**, **Z**), the *β* estimator from different methods would be measuring different effects (total or direct effects) or would even be invalid. Therefore, as already mentioned, the results of ADNI analysis in this paper simply serve as a demonstration of our *CIV* methods, and must not be used to make definite causal statements regarding AD.

In future research we will attempt to overcome some of the limitations of the *CIV* methods. One useful direction is to propose a measure of quality of solutions. Such a measure could be used to discard solutions of poor quality, or alternatively to combine solutions using quality based weights. A more complex approach could also be developed by adding further soft constraints (e.g., quality based constraints, group constraints) to the current version of our *CIV_smooth* algorithm.

In future work we intend to apply our approach to study causation in a larger setting. Such data could be obtained from UKbiobank (Sudlow et al., 2015), which is a prospective data containing health information of 500,000 participants as well as their genetic profiles. UKbiobank is an ideal source to study the causal effects of multiple potentially pleiotropic biomarkers, since it contains information on a rich variety of phenotypes and disease outcomes (including AD) for each participant. However, the large sample size of the UKbiobank presents a serious computational challenge for *CIV_smooth*. We need, therefore, to develop successful strategies to integrate MR results from subsamples of workable size for *CIV_smooth*.

In conclusion, this paper proposes a new approach (*CIV_smooth*) for conducting MR analyses when pleiotropy is suspected. Assuming a linear structural model linking together genotypes, phenotypes, and outcome yields “approximately valid” instruments to adjust causal effect estimation when potential pleiotropic phenotypes are measured. We have shown in simulations that the performance of (*CIV_smooth*) is comparable, and occasionally preferable, to other popular methods, namely *2SLS*, *Allele*, and *sisVIVE*. We have also shown in the analysis of a data set on AD that the method produces reasonable results. In view of these results, we hope that *CIV_smooth* will be integrated into the family of MR analyses methods, making MR a more common practice even when pleiotropy is observed.

## Supporting information

Supporting information
