# Supplementary material for: Constrained instruments and their application to Mendelian randomization with pleiotropy
Source: Genet Epidemiol. 2019 Jan 12;43(4):373–401. doi: 10.1002/gepi.22184 (PMC6537099; doi:10.1002/gepi.22184)
Supplement: Supplementary file 1 — Supporting information [file GEPI-43-373-s001.pdf]

Supplemental Materials: Constrained Instruments and their Application to Mendelian Randomization with Pleiotropy

Lai Jiang, Karim Oualkacha, Vanessa Didelez, Antonio Ciampi, Pedro Rosa,

Andrea L. Benedet, Sulantha Mathotaarachchi, Brent Richards,

Celia M.T. Greenwood *

A Controlling Z as Covariates or as Exogenous variables

Consider the following two ways to control **Z** in TSLS:

(1) The *2SLS* estimate of β_1 (causal effect of **X** on **Y**), by adjusting **Z** as covariates, is obtained from the regression models:

$$\mathbf{X} = \alpha_1 \mathbf{G} + \alpha_2 \mathbf{Z} + u, \tag{1a}$$

$$\mathbf{Y} = \beta_1 \hat{\mathbf{X}} + \beta_2 \mathbf{Z} + v, \tag{1b}$$

where $\hat{\mathbf{X}}$ is the fitted value from first stage regression (1a), i.e. $\hat{\mathbf{X}} = \widehat{\alpha_1}\mathbf{G} + \widehat{\alpha_2}\mathbf{Z}$. We will call this estimate $\hat{\beta}_1$.

(2) The "*2SLS_exo*" estimate of β_1, by treating **Z** as exogenous variables, is obtained from the

*Corresponding Author. Email: celia.greenwood@mcgill.ca

regression models:

$$(\mathbf{I}-\mathbf{P_z})\mathbf{X} = \alpha_1^*(\mathbf{I}-\mathbf{P_z})\mathbf{G} + (I-P_z)u, \tag{2a}$$

$$(\mathbf{I}-\mathbf{P_z})\mathbf{Y} = \beta_1^*\hat{\mathbf{X}}^* + v_1. \tag{2b}$$

where $\mathbf{P_z} = \mathbf{Z}^\top(\mathbf{Z}^\top\mathbf{Z})^{-\mathbf{1}}\mathbf{Z}$ and $\hat{\mathbf{X}}^*$ is the fitted value from first stage regression (2a), i.e. $\hat{\mathbf{X}}^* = \widehat{\alpha}_1^*(\mathbf{I}-\mathbf{P_z})\mathbf{G}$. We will call this estimate $\hat{\beta}_1^*$.

Now we prove $\hat{\beta}_1 = \hat{\beta}_1^*$. Indeed, (1a) and (2a) imply $\widehat{\alpha_1} = \widehat{\alpha}_1^*$ according to Frisch–Waugh–Lovell theorem (Frisch and Waugh (1933), Lovell (2008)). Then we have $\hat{\mathbf{X}}^* = \widehat{\alpha}_1^*(\mathbf{I}-\mathbf{P_z})\mathbf{G} = \widehat{\alpha}_1(\mathbf{I}-\mathbf{P_z})\mathbf{G} = (\mathbf{I}-\mathbf{P_z})\hat{\mathbf{X}}$, and multiplying both sides of (1b) by $(\mathbf{I}-\mathbf{P_z})$, we finally have

$$(\mathbf{I}-\mathbf{P_z})\mathbf{Y} = (I-P_z)\beta_1\hat{\mathbf{X}} + (I-P_z)v = \beta_1\hat{\mathbf{X}}^* + (I-P_z)v. \tag{3a}$$

Therefore, comparing (2b) and (3a) we obtain $\hat{\beta}_1 = \hat{\beta}_1^*$.

B Solution to the Constrained Instrumental Variable Problem

Let $\mathbf{M} = \mathbf{X}(\mathbf{X}^\top\mathbf{X})^{-\mathbf{1}}\mathbf{X}^\top$, then $\mathbf{c}^\top\mathbf{G}^\top\mathbf{X}\mathbf{v} = \mathbf{c}^\top\mathbf{G}^\top\mathbf{X}(\mathbf{X}^\top\mathbf{X})^{-\mathbf{1}}\mathbf{X}^\top\mathbf{X}\mathbf{v} = \mathbf{c}^\top\mathbf{G}^\top\mathbf{M}\mathbf{X}\mathbf{v}$,

$$\max_{\mathbf{c}\in\mathbf{R}^\mathbf{p},\mathbf{v}\in\mathbf{R}^\mathbf{r}} \mathbf{c}^\top\mathbf{G}^\top\mathbf{X}\mathbf{v} = \max_{\mathbf{c}\in\mathbf{R}^\mathbf{p},\mathbf{v}\in\mathbf{R}^\mathbf{r}} \mathbf{c}^\top\mathbf{G}^\top\mathbf{M}\mathbf{X}\mathbf{v} \leq ||\mathbf{c}^\top\mathbf{G}^\top\mathbf{M}||\ ||\mathbf{X}\mathbf{v}||,$$

where $||\mathbf{X}\mathbf{v}|| = \sum_{i=1}^{n} |(\mathbf{X}\mathbf{v})_i|^2$ denotes the norm of vector $\mathbf{X}\mathbf{v}$ in the inner product space R^p. The equality holds if and only if $\mathbf{X}\mathbf{v}$ and $\mathbf{M}\mathbf{G}\mathbf{c}$ are collinear. Let $\mathbf{w} = (\mathbf{G}^\top\mathbf{G})^{\frac{1}{2}}\mathbf{c}$ then the problem is equivalent to

$$\max_{\mathbf{w}\in\mathbf{R}^\mathbf{p}} \mathbf{w}^\top\mathbf{A}\mathbf{w}$$

subject to conditions:

$$\mathbf{w}^\top\mathbf{w} = 1$$

$$\mathbf{B}^\top\mathbf{w} = \mathbf{0}$$

where $\mathbf{A} = (\mathbf{G}^\top\mathbf{G})^{-\frac{1}{2}}\mathbf{G}^\top\mathbf{M}\mathbf{G}(\mathbf{G}^\top\mathbf{G})^{-\frac{1}{2}}$ and $\mathbf{B} = (\mathbf{G}^\top\mathbf{G})^{-\frac{1}{2}}\mathbf{G}^\top\mathbf{Z}$.

If we have $\text{rank}(\mathbf{A}) = p \geq \text{rank}(\mathbf{Z}) = k$ (columns are uncorrelated), then there exists a solution for $\mathbf{w}$ since this is a quadratic optimization problem with quadratic/linear constraints (Golub, 1973).

Consider the QR decomposition of $\mathbf{B}$:

$$\mathbf{B} = \mathbf{Q}^\top \begin{bmatrix} \mathbf{R} & \mathbf{S} \\ 0 & 0 \end{bmatrix} \blacksquare \tag{4}$$

where $\mathbf{R}$ is a k by k upper triangular matrix with positive diagonal elements. $\mathbf{Q}$ is an orthogonal matrix. $\mathbf{S}$ is a k by $p-k$ matrix and $\blacksquare$ represents the column permutation matrix (Gu and Eisenstat, 1996) to ensure that the diagonal elements of $\mathbf{R}$ are positive and non-increasing, i.e. $\mathbf{R}$ is invertible. $\mathbf{R}$ is then unique under these conditions (Golub and Van Loan, 1996).

Now we let $\mathbf{w} = \mathbf{Q}^\top \begin{pmatrix} \mathbf{t} \\ \mathbf{d} \end{pmatrix}$ where $\mathbf{t} \in R^k, \mathbf{d} \in R^{p-k}$ and $\mathbf{QAQ}^\top = \begin{bmatrix} \mathbf{A}_{1,1} & \mathbf{A}_{1,2} \\ \mathbf{A}_{2,1} & \mathbf{A}_{2,2} \end{bmatrix}$.

The problem then becomes:

$$\max_{\mathbf{d} \in \mathbf{R}^{(\mathbf{p}-\mathbf{k})}} \mathbf{d}^\top \mathbf{A}_{\mathbf{2},\mathbf{2}}^\top \mathbf{d} \tag{5}$$

subject to conditions:

$$\mathbf{d}^\top \mathbf{d} = 1$$

We now know that the solution for $\mathbf{d}$ is any eigenvector corresponding to the largest eigenvalue of $\mathbf{A}_{2,2}$. There are at most $p-k$ of them.

In conclusion, when $n > p$ the (unique) solution of the constrained instrumental variable (*CIV_naive*) is $\mathbf{Gc} = \mathbf{G}(\mathbf{G}^\top \mathbf{G})^{-\frac{1}{2}} \mathbf{Q}^\top \begin{pmatrix} 0 \\ \mathbf{d} \end{pmatrix}$, where $\mathbf{Q}$ is an orthogonal matrix defined by (4) and $\mathbf{d}$ is an eigenvector defined by (5).

C Algorithm for obtaining approximately sparse constrained CIV solutions with L_0 penalty

1. Initialization: For a given value of λ, start from an initial guess $\tilde{\mathbf{c}}$ and initial L_0 penalty $\sigma_{\max} = \max_j |\tilde{c}_j|$, set $\sigma = \sigma_{\max}$.

2. While $\sigma > \sigma_{\min} = 0.01$ we do

   i Calculate the gradient of function (6) $\mathbf{d} \in \mathbb{R}^p$, where $d_j = \frac{\lambda \tilde{c}_j}{\sigma^2} \exp(-\frac{\tilde{c}_j^2}{2\sigma^2}) - 2[\tilde{\mathbf{c}}^\top \mathbf{G}^\top \mathbf{M} \mathbf{G}]_j, j \in \{1, ..., p\}$ and $\mathbf{M} = \mathbf{X}(\mathbf{X}^\top \mathbf{X})^{-\mathbf{1}} \mathbf{X}^\top$.

   ii Set $\mathbf{c} = (\mathbf{I} - \mathbf{A}^- \mathbf{A})(\tilde{\mathbf{c}} - \mu \mathbf{d})$ where $\mathbf{A}^-$ is a generalized inverse of $\mathbf{A} = \mathbf{Z}^\top \mathbf{G}$ and μ is a step-size parameter in gradient descent algorithm.

   iii Set $\mathbf{c}^* = \mathbf{c}/\sqrt{\mathbf{c}^\top \mathbf{G}^\top \mathbf{G} \mathbf{c}}$ as the updated solution.

   iv Repeat (i) (ii) and (iii) (maximum T times) until it converges, i.e. $\sqrt{\sum_{j=1}^{p} (|\mathbf{c}_\mathbf{j}^*| - |\mathbf{c}_j|)^2 / p} < 10^{-10}$.

3. Update σ with $\sigma = 0.5\ \sigma_{\text{prev}}$, where σ_{prev} is the previous value of σ used in step 2. If $\sigma > \sigma_{\min}$ repeat all items in step 2. If not, stop the algorithm and record the last iteration of $\mathbf{c}$ as the final solution.

The maximization problem of Equation (8) is solved by repeatedly taking gradient descent steps (i), and then projecting the possible solution back into constrained set ((ii),(iii)). The step (ii) restricts the solution to be on the constrained set (7b) and step (iii) restricts it to the boundary of the constrained set (7a). The unconstrained gradient descent step followed by projection to the feasible set is equivalent to a direct gradient descent step on the feasible set (Cui et al., 2010). The parameters for step-size (μ) and number of iterations (T) should be carefully chosen to achieve balance between computation cost and precision. That is, the states discovered by this algorithm may not achieve the global maximum value of Equation (6) even with a large number of iterations

if we use a step size that is too large. The decreasing list of values for σ is chosen to ensure that the approximation accuracy will gradually increase.

D Non-unique Solutions to the *CIV_smooth* Problem

There may be multiple local solutions to the smoothed problem (Eq 8), which is not a convex optimization problem. Note that the maximization of a convex function over a convex set is not necessarily a convex problem. As a result, a local maximum solution of **c** may not be the global maximum solution, and numerical optimization techniques may get trapped into a local maximum. Therefore, we start from multiple (e.g. 100) initial points randomly sampled from a multivariate normal distribution $N(0, I_p)$, and let the smoothed L_0 algorithm converge to a set of solutions, possibly arriving at multiple local modes. After examining correlations between all pairs of solutions, highly correlated solutions (≥ 0.9) are removed. The remaining solutions are combined into a matrix $\mathbf{c}^*$ (of p rows). Finally, we construct new instruments $\mathbf{G}\mathbf{c}^*$ and refer to them as the *CIV_smooth* instruments.

An example of this potential multi-modal problem is shown in Figure S1, where one simulated dataset from Series II was analyzed. The hierarchical cluster dendrogram shows the solution space for this simulation, demonstrating that there exist multiple different *CIV_smooth* solutions. However, a principal component analysis of the solutions shows that in most simulations only 1 unique solution stands out. So although multiple distinct solutions do occur, they tend to be similar to each other. To obtain our *CIV_smooth* estimator, we sample possible solutions by starting our converging iterations from multiple initial points, and combining all distinct solutions into a matrix $\mathbf{c}^*$. This approach provides a set of the potential instruments with strong association with $\mathbf{X}$ and low correlation with $\mathbf{Z}$.

Figure S1: Cluster dendrogram (a) and principal component analysis (b) from a one-sample set-up, with $\mathbf{X} \rightarrow \mathbf{Z}$ and $\alpha_x = \alpha_z = 0.1$ across 200 simulations.

(a) Cluster dendrogram of all (100) converged *CIV_smooth* solutions in one simulation. Red block denotes the identified hierarchical clusters using the number of clusters determined by the silhouette coefficient (Rousseeuw, 1987).

(b) Top: The proportion of the top eigenvalue (among all values) of all *CIV_smooth* solutions, across 200 simulations. Bottom: The total number of principal components (with eigenvalue $\geq$ 0.8), across 200 simulations.

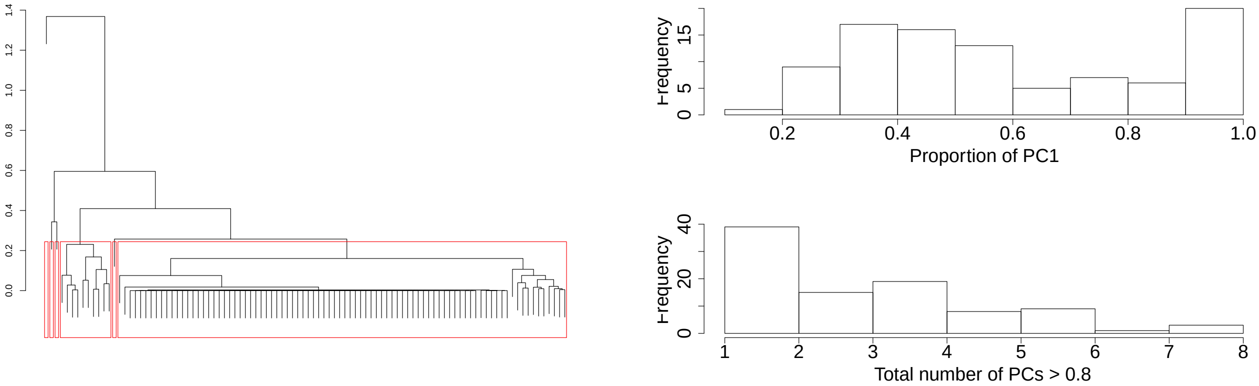

E Choice of the Tuning Parameter λ

The tuning parameter λ controls the amount of regularization, and each value of it corresponds to a specific fitted model. In general, the value of tuning parameter in regularization is chosen to achieve (i) prediction accuracy and (ii) recovering the valid model. The first goal is straightforward and can be easily attained by optimizing the prediction error $\mathbf{Y} - \mathbf{X}\beta^*$. The latter is more important for our approach in the existence of pleiotropy because pleiotropic genotypes could be "informative" for a prediction model, but render the whole MR analysis invalid. Thus we choose the projected prediction error (Kang et al., 2016), $||\mathbf{P}_{\mathbf{G}^*}(\mathbf{Y} - \mathbf{X}\beta^*)||$, instead of the prediction error, $\mathbf{Y} - \mathbf{X}\beta^*$, as the measure to obtain optimal tuning parameter value λ. Moreover, 10-fold cross-validation is employed to estimate the target projected prediction error on a given dataset, in order to obtain consistent choice of λ.

We demonstrate the advantage of choosing λ according to minimized projected prediction error in *CIV_smooth* with a simple simulation. The simulated data contains 9 invalid SNPs and 1 valid SNP, and *CIV_smooth* is implemented on this data to obtain coefficient u_j for each SNP j on

different values of λ. The regularization path of *CIV_smooth* (the solution values of coefficient **c** with respect to λ), is demonstrated in Figure S2. Notice that the coefficient of invalid SNPs could grow with increasing levels of regularization at the beginning of the path ($0 \rightarrow 0.1$). Moreover, $\hat{\lambda}_2$, the tuning parameter value corresponding to minimized projected prediction error leads to a more sparse solution **c** than $\hat{\lambda}_1$, which corresponds to minimized prediction error.

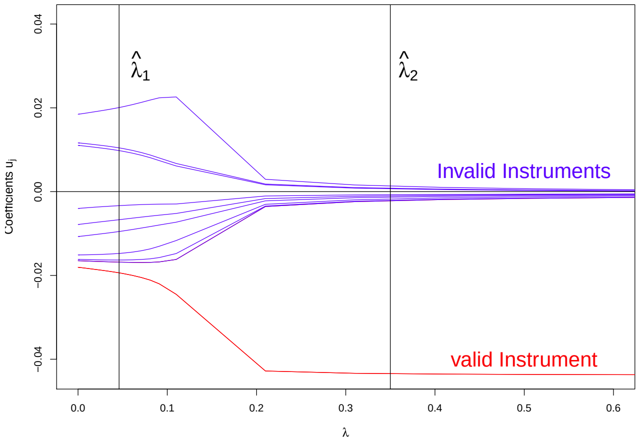

Figure S2: Illustration of regularization path for *CIV_smooth* on simulated data with 10 genotypes ($p = 10$), in which only one genotype is a valid instrument. Blue lines: the coefficient paths of 9 invalid instruments. Red line: the coefficient path of the only valid instrument. X axis: increasing values of λ corresponding to increasing levels of regularization. Y axis: coefficient value u_j for each SNP j. $\hat{\lambda}_1$ vertical line: the choice of λ that minimizes the prediction error. $\hat{\lambda}_2$ vertical line: the choice of λ that minimizes the projected prediction error.

F Sensitivity analysis:

Z Contains Only Part of Pleiotropic Phenotypes

The purpose of this section is to evaluate the performance of *CIV_smooth* method and its competitors, as we vary the proportions of observed pleiotropic phenotypes included in **Z**. Our simulation design follows the structure equations in Section 4.1, including both Series I ($\gamma_{xz} = \gamma_{zx} = 0$) and series II ($\gamma_{zx} \neq 0$). Also, the association parameters α_Z were varied to study the impact of strong ($\alpha_z = 1$) or weak ($\alpha_z = 0.1$) pleiotropic effects on performance. In each of the $2 \times 2 = 4$ scenarios described above, the number ($p_z = 20$ or 50) of genotypes associated with **Z** and the proportion (0.1, 0.4, 0.6, 0.9) of pleiotropic phenotypes observed were varied to assess *CIV_smooth* performance. We adopt one-sample set-up, as described in Section 2.5, in all simulations.

Specifically, we simulated a set of independent genotypes **G** in the same way as Section 4.1. (**X**, **Z** **Y**) were simulated using the following equations:

Simulation Series I : Standard Pleiotropy

$$
\begin{aligned}
x_i &= \alpha_x \sum_{j=1}^{p} G_{ij} + u_i + \varepsilon_{x,i}, \\
z_{ik}^* &= \alpha_z G_{ik} + u_i + \varepsilon_{z,k,i}, \;\; k = 1, ..., p_z, \\
y_i &= x_i + \sum_{k=1}^{p_z} z_{ik}^* + u_i + \varepsilon_{y,i}, \\
S_z &\subset (1, ..., p_z), \quad \mathbf{Z_i} = \{z_{ik}^*\}, k \in S_z,
\end{aligned}
\tag{6}
$$

where $\varepsilon_{x,i}, \varepsilon_{z,k,i}, \varepsilon_{y,i}, u_i \sim N(0,1)$. $\mathbf{Z_i^*} = (z_{i1}^*, ..., z_{ip_z}^*)$ is the set of all pleiotropic phenotypes for ith sample. S_z is a randomly selected subset of $(1, ..., p_z)$ corresponding to a proportion of all pleiotropic phenotypes ($|S_z|/p_z \in \{0.1, 0.4, 0.6, 0.9\}$). $\mathbf{Z_i}$ contains the correspondingly observed pleiotropic phenotypes with respect to S_z. See Table 2 for details about the rest parameters ($\alpha_x, \alpha_z, n, p, p_z$).

Simulation Series II : $\mathbf{Z} \rightarrow \mathbf{X}$

$$
\begin{aligned}
z_{ik}^{*} &= \alpha_z G_{ik} + u_i + \varepsilon_{z,k,i}, \ \ k = 1, ..., p_z, \\
x_i &= \alpha_x \sum_{j=1}^{p} G_{ij} + \sum_{k=1}^{p_z} z_{ik}^{*} + u_i + \varepsilon_{x,i}, \\
y_i &= x_i + \sum_{k=1}^{p_z} z_{ik}^{*} + u_i + \varepsilon_{y,i}, \\
S_z &\subset (1, ..., p_z), \quad \mathbf{Z_i} = \{z_{ik}^{*}\}, k \in S_z,
\end{aligned}
\tag{7}
$$

where $\varepsilon_{x,i}, \varepsilon_{z,k,i}, \varepsilon_{y,i}, u_i \sim N(0,1)$.

We conducted one-sample simulations for each scenario from series I or II, with varying values of α_z, p_z and proportions of pleiotropic phenotypes observed. In each simulation we compared the bias of causal effect estimators across all the methods (used in the Section 4), and show the results in Figures S3 - S6. The graphs demonstrate that *sisVIVE* and *sisVIVE_exo* methods yield the best causal effect estimation among all competitors in most scenarios. However, if $\alpha_z = 0.1$ and proportion of observed pleiotropic phenotypes are more than 50% , then *CIV_smooth* can provide better causal effect estimation than *sisVIVE* methods and *Allele* methods.

	$p_z = 20$	$p_z = 50$
Observed Proportion 0.1	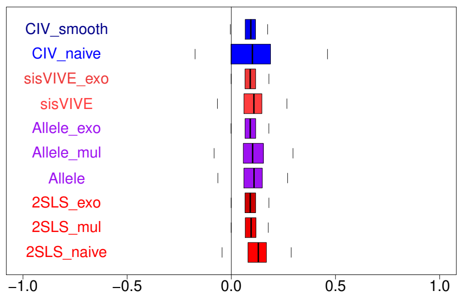	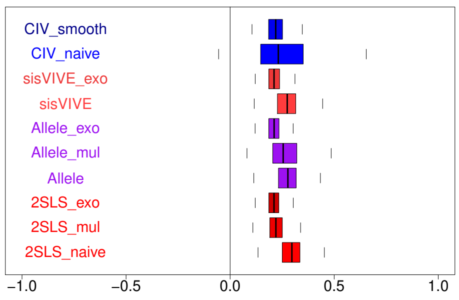
Observed Proportion 0.4	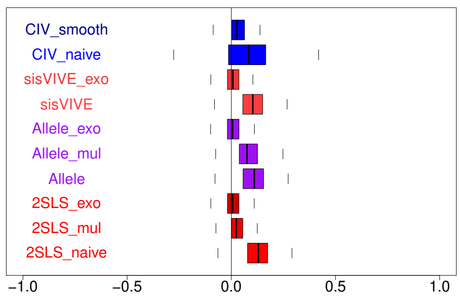	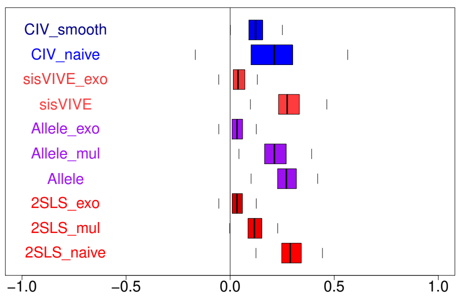
Observed Proportion 0.6	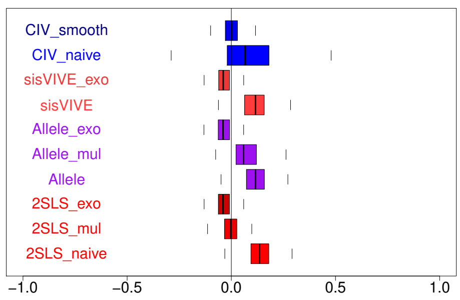	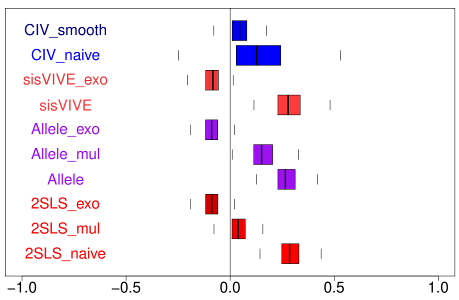
Observed Proportion 0.9	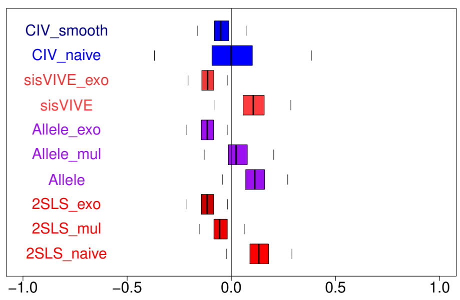	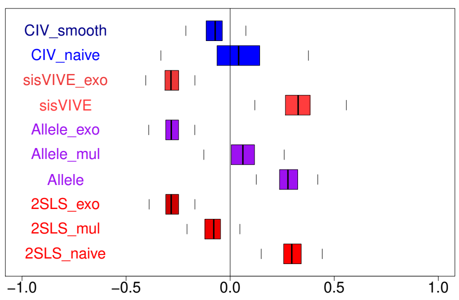

Figure S3: Sensitivity analysis results for proportion of pleiotropic phenotypes observed. Boxplots of the bias of the causal effect estimates, $\hat{\beta} - 1$, are shown using the same parameter settings as in simulation Series I, in the scenario of weak pleiotropy ($\alpha_x = 1, \alpha_z = 0.1$). The rows display results corresponding to different values of the proportion of observed pleiotropic phenotypes. the columns show the number of genotypes associated with $\mathbf{Z}$ out of 100 genotypes associated with $\mathbf{X}$.

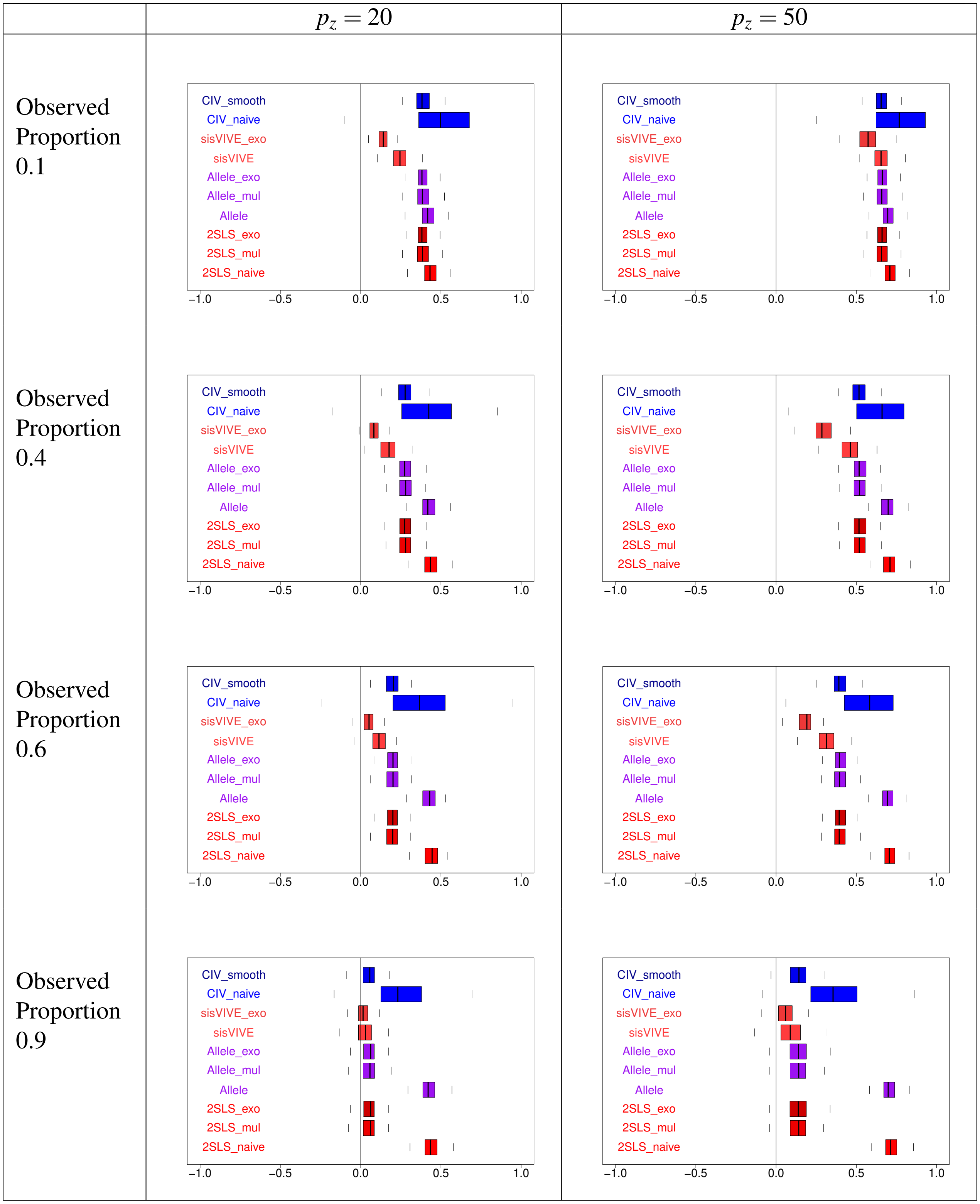

Figure S4: Sensitivity analysis results for proportion of pleiotropic phenotypes observed. Boxplots of the bias of the causal effect estimates, $\hat{\beta} - 1$, are shown using the same parameter settings as in simulation Series I, in the scenario of strong pleiotropy ($\alpha_x = 1, \alpha_z = 1$). The rows display results corresponding to different values of the proportion of observed pleiotropic phenotypes. the columns show the number of genotypes associated with $\mathbf{Z}$ out of 100 genotypes associated with $\mathbf{X}$.

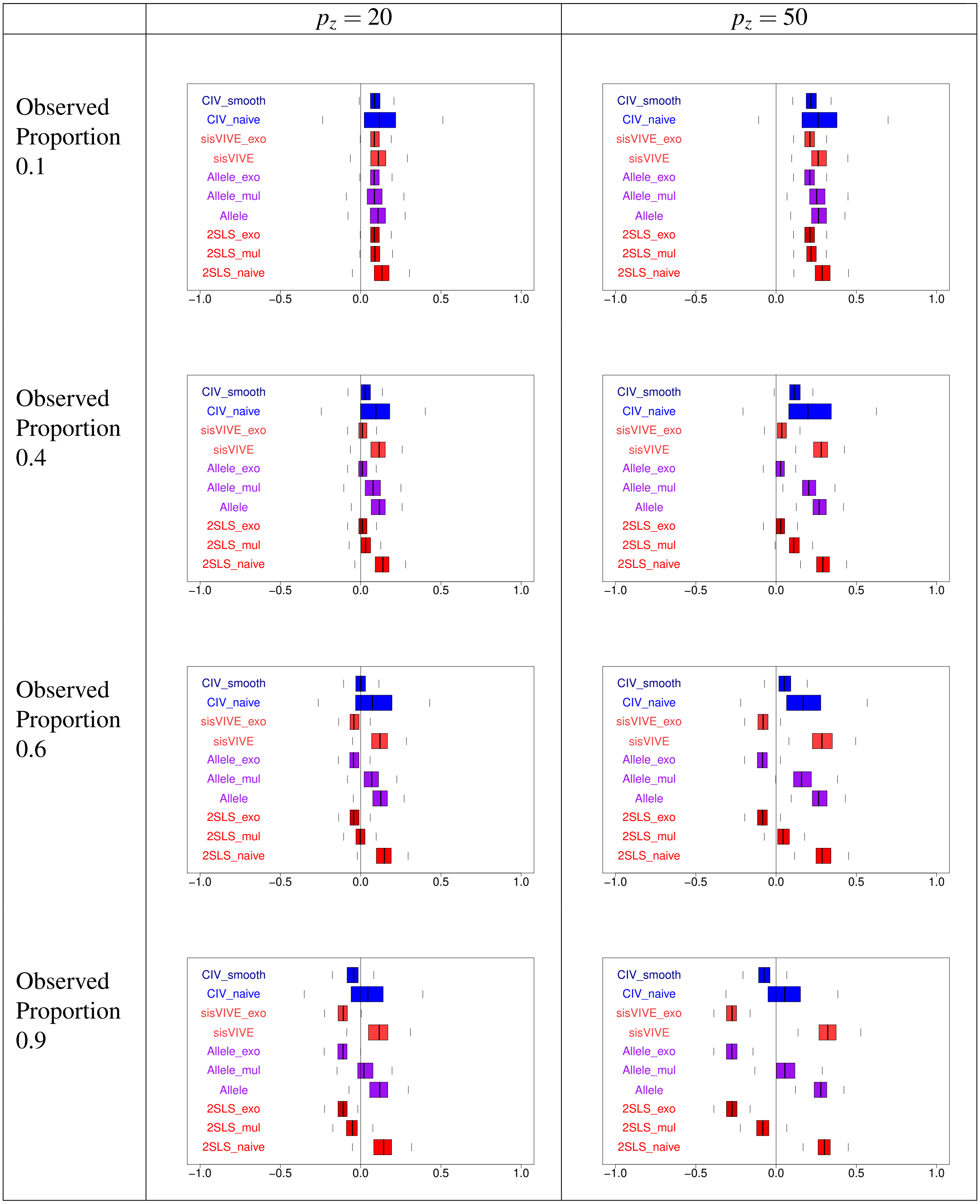

Figure S5: Sensitivity analysis results for proportion of pleiotropic phenotypes observed. Boxplots of the bias of the causal effect estimates, $\hat{\beta} - 1$, are shown using the same parameter settings as in simulation Series II, in the scenario of weak pleiotropy ($\alpha_x = 1, \alpha_z = 0.1$). The rows display results corresponding to different values of the proportion of observed pleiotropic phenotypes. the columns show the number of genotypes associated with $\mathbf{Z}$ out of 100 genotypes associated with $\mathbf{X}$.

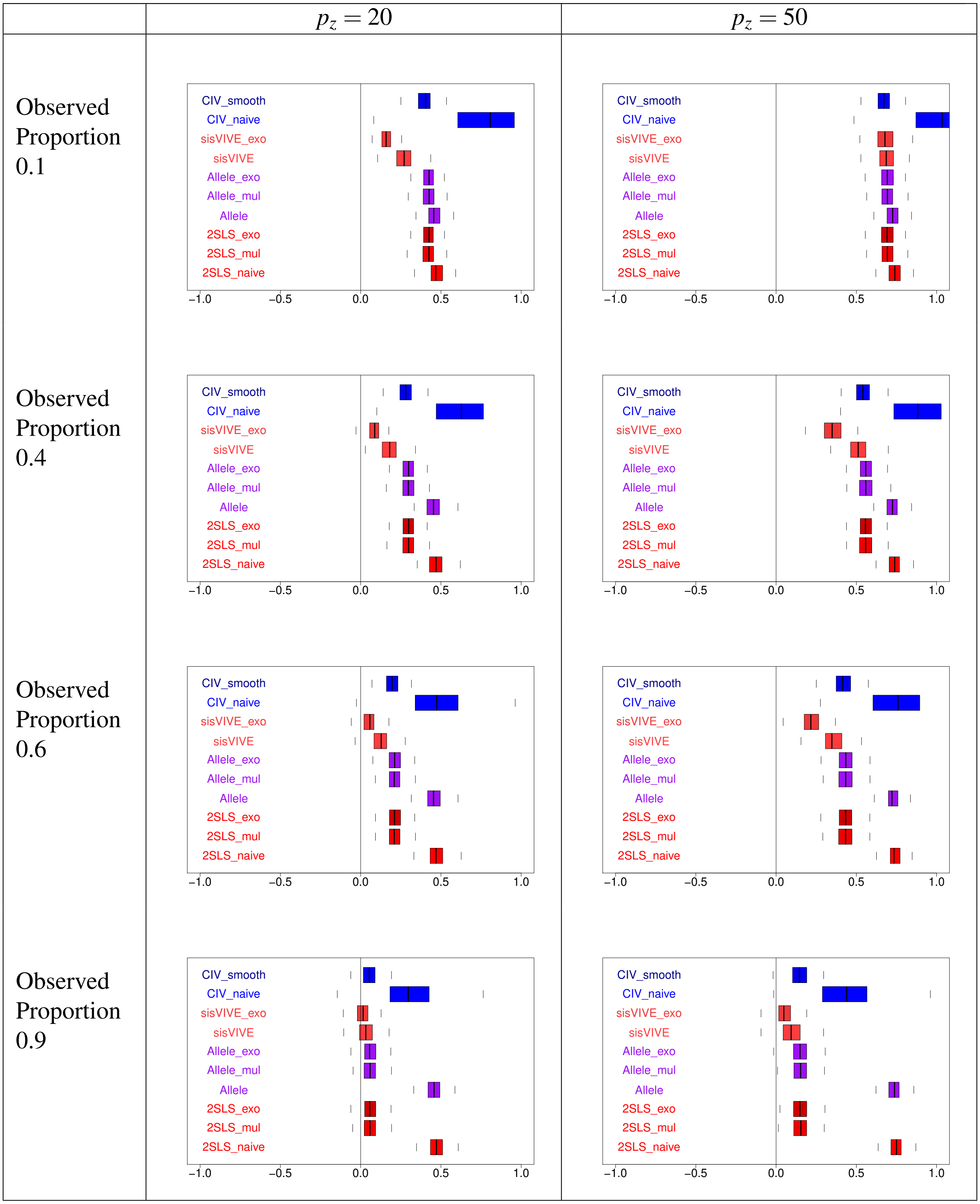

Figure S6: Sensitivity analysis results for proportion of pleiotropic phenotypes observed. Boxplots of the bias of the causal effect estimates, $\hat{\beta} - 1$, are shown using the same parameter settings as in simulation Series II, in the scenario of strong pleiotropy ($\alpha_x = 1, \alpha_z = 1$). The rows display results corresponding to different values of the proportion of observed pleiotropic phenotypes. the columns show the number of genotypes associated with $\mathbf{Z}$ out of 100 genotypes associated with $\mathbf{X}$.

References

Zhifu Cui, Hang Zhang, and Wei Lu. An improved smoothed l0-norm algorithm based on multiparameter approximation function. In *Communication Technology (ICCT), 2010 12th IEEE International Conference on*, pages 942–945. IEEE, 2010.

Ragnar Frisch and Frederick V Waugh. Partial time regressions as compared with individual trends. *Econometrica: Journal of the Econometric Society*, pages 387–401, 1933.

Gene H Golub. Some modified matrix eigenvalue problems. *Siam Review*, 15(2):318–334, 1973.

Gene H Golub and Charles F Van Loan. Matrix computations. 1996. *Johns Hopkins University, Press, Baltimore, MD, USA*, pages 374–426, 1996.

Ming Gu and Stanley C Eisenstat. Efficient algorithms for computing a strong rank-revealing qr factorization. *SIAM Journal on Scientific Computing*, 17(4):848–869, 1996.

Hyunseung Kang, Anru Zhang, T Tony Cai, and Dylan S Small. Instrumental variables estimation with some invalid instruments and its application to mendelian randomization. *Journal of the American Statistical Association*, 111(513):132–144, 2016.

Michael C Lovell. A simple proof of the fwl theorem. *The Journal of Economic Education*, 39(1): 88–91, 2008.

Peter J Rousseeuw. Silhouettes: a graphical aid to the interpretation and validation of cluster analysis. *Journal of computational and applied mathematics*, 20:53–65, 1987.
